# Hippocampal neuroinflammation and altered peripheral neurobiological protein profile in experimental arthritis and systemic juvenile idiopathic arthritis

**DOI:** 10.1016/j.ebiom.2026.106330

**Published:** 2026-06-16

**Authors:** Xingzhao Wen, Heshuang Qu, Malgorzata Benedyk-Machaczka, Daphne Chen, Erik Sundberg, Erik Melén, Maria Altman, Cecilia Aulin, Helena Erlandsson Harris

**Affiliations:** a Division of Rheumatology, Department of Medicine Solna, Center for Molecular Medicine, Karolinska Institutet, Stockholm, Sweden; b Department of Microbiology, Faculty of Biochemistry, Biophysics and Biotechnology, Jagiellonian University, Krakow, Poland; c Unit of Paediatric Rheumatology, Karolinska University Hospital, Stockholm, Sweden; d Department of Women's and Children's Health, Karolinska Institutet, Stockholm, Sweden; e Centre for Occupational and Environmental Medicine, Region Stockholm, Stockholm, Sweden; f Broegelmann Research Laboratory, Department of Clinical Science, University of Bergen, Bergen, Norway

**Keywords:** Juvenile idiopathic arthritis, Neuroinflammation, Oxidative stress, Biomarker, Collagen-induced arthritis, HMOX2

## Abstract

**Background:**

Children with juvenile idiopathic arthritis (JIA) are reported to exhibit increased rates of symptoms affecting emotional regulation and behaviour. However, underlying biological mechanisms remain unclear. Inflammation in the central nervous system (CNS) can be triggered by peripheral immune effects and may contribute to these observations. In this study, we aimed to investigate whether neurobiological alterations are present in systemic JIA (sJIA), whether CNS inflammation occurs during arthritis, and the potential underlying mechanisms.

**Methods:**

Plasma samples from patients with active sJIA (n = 16) and sex- and age-matched healthy controls (HCs, n = 16), together with paired samples from the same patients with sJIA during inactive disease (n = 12), were analysed using Olink proteomics to determine peripheral neurobiological and inflammation protein profiles. Clinical data was retrieved from the Swedish Paediatric Rheumatology Register and medical charts. CNS neuroinflammatory responses and underlying mechanisms were further investigated using *in vivo* and *in vitro* experiments.

**Findings:**

Patients with active sJIA exhibited altered plasma neurobiological protein profiles compared with HCs. These alterations correlated with higher scores of pain and life impact in patients, suggesting that the altered profiles may reflect neurofunctional changes. Notably, the neurobiological protein profiles remained altered even during inactive phases of the disease. In chronic arthritic mice, microglial activation and impaired neurogenesis were observed in the hippocampus, with no significant cortical changes. RNA-seq analysis implicated mitochondrial dysfunction and oxidative stress in mediating neuroinflammation during chronic arthritis in mice. Haem oxygenase 2 (HMOX2) was identified as a peripheral biomarker indicating microglial activation in the hippocampus (Spearman r = −0.886, *P* = 0.019). Combined neurobiological and inflammation profiling in patients with sJIA implicated interleukin-6 (IL-6) and interleukin-18 (IL-18) as potential drivers of hippocampal microglial activation during arthritis.

**Interpretation:**

Our findings highlight the effects of sJIA and experimental arthritis on the nervous system and provide insights for clinical monitoring and the development of targeted therapeutic strategies.

**Funding:**

This study was funded by grants from the Swedish Research Council and The Swedish Rheumatism Association.

**Research in context**

**Evidence before this study**

We searched PubMed up to February 2026 using the following terms: (“juvenile idiopathic arthritis” OR “rheumatoid arthritis” OR “arthritis”) AND (“psychiatric disorder” OR “emotional disorder” OR “cognition” OR “neuroinflammation” OR “neurogenesis”). Increasing evidence indicates that children with juvenile idiopathic arthritis (JIA) experience higher rates of emotional and behavioural disturbances compared with their healthy peers. Systemic inflammation and chronic arthritis are suspected to affect the central nervous system, but biological mechanisms in systemic JIA (sJIA) are poorly understood.

**Added value of this study**

In this study, we demonstrate that patients with sJIA have a distinct plasma neurobiological protein profile compared with healthy controls, which correlate with higher pain and life impact scores. In chronic arthritic mice, hippocampal microglial activation, impaired neurogenesis, and mitochondrial dysfunction with oxidative stress are presented. By combining patient and mouse data, we identify haem oxygenase 2 (HMOX2) as a candidate plasma biomarker of hippocampal neuroinflammation and implicate IL-6, and especially IL-18, as key mediators linking chronic arthritis to neurobiological changes.

**Implications of all the available evidence**

This study provides molecular evidence of neurobiological alterations in patients with sJIA and supports incorporating neurobiological and neuropsychiatric monitoring into the clinical follow-up of children with sJIA. We highlight the mechanistic targets and measurable biomarkers (e.g., HMOX2) for future studies and trials aiming to modulate neuroinflammation in chronic arthritis. This study may inform the development of personalised treatment strategies, including IL-18–directed therapies, for patients at risk of neurological or psychosocial complications.

## Introduction

Juvenile idiopathic arthritis (JIA) is the most common chronic rheumatic disease in children under 16 years of age, characterised by persistent joint inflammation and significant impact on quality of life.^1^^,^^2^ JIA consists of seven subtypes defined by the International League of Associations for Rheumatology (ILAR).^3^ Although the clinical features vary among JIA subtypes, all patients exhibit inflammation, joint destruction, pain, and fatigue.^3^ The subtype systemic JIA (sJIA) is the most severe form of JIA, with flares of systemic inflammation in addition to arthritis.^4^

Beyond musculoskeletal symptoms, clinicians have also observed psychiatric impairment in patients with JIA.^5^, ^6^, ^7^, ^8^, ^9^ Children with JIA have been reported to experience higher rates of depression and anxiety compared to their healthy peers.^5^^,^^8^^,^^9^ Lower scores in neurocognitive performance tests were reported in JIA patients as compared with age-matched healthy individuals.^7^ In addition, as adults, these patients continue to display cognitive differences relative to the general population, which can significantly impact their work and daily life and may persist throughout their lifetime.^10^^,^^11^ For JIA patients, psychiatric and cognitive impairments are commonly associated to factors like chronic pain, social limitations and restricted physical activities.^6^^,^^8^^,^^12^ Recently, a few studies have suggested that neuroinflammation in the CNS may occur in patients with JIA and could contribute to psychiatric dysfunction.^13^^,^^14^ Despite these observations, the pathological changes in the CNS associated with JIA remain poorly understood. Furthermore, the mechanisms underlying CNS involvement in JIA remain largely unknown. Currently, there are no specific treatment or biomarkers available for neuropsychiatric manifestations in JIA.

Neuroinflammation, inflammation in the CNS, has been identified as a contributing factor to cognitive decline and psychiatric disorders.^15^^,^^16^ Neuroinflammation has been suggested to precipitate alterations in neurotransmitter systems, leading to behavioural and cognitive changes.^17^ During neuroinflammation, resident CNS cells such as microglia, astrocytes, and oligodendrocytes become activated and contribute to the inflammatory response by releasing cytokines, chemokines, damage-associated molecular patterns (DAMPs), and reactive oxygen species (ROS), which in turn can damage brain tissue and influence the onset of psychiatric manifestations.^18^^,^^19^ More importantly, increased evidence suggests that neuroinflammation can be triggered by peripheral effectors, such as autoimmune diseases, infection, and surgery.^20^^,^^21^

In this study, we collected plasma samples from patients with sJIA to investigate whether the profile of neurobiologically related proteins is altered compared with that of healthy children. In parallel, we used a chronic arthritis mouse model to explore if CNS neuroinflammation presents during chronic arthritis and to investigate underlying mechanisms linking arthritis and neuroinflammation.

## Methods

### Clinical study population

Plasma samples were collected from 16 patients with sJIA at Astrid Lindgren's Children's Hospital, Karolinska University Hospital, Stockholm, Sweden (sample collection JABBA, 2010–2019). 16 age- and sex-matched healthy individuals (Barnens Miljö-och Hälsoundersökning cohort) from the Stockholm region were selected and were matched to patients on a one-to-one basis by sex assigned at birth and age category. As the healthy control cohort included children aged 4, 8, and 12 years, exact age matching was not feasible for all patients. Therefore, patients with sJIA were grouped into predefined age bands (1–5 years, 6–10 years, and ≥11 years), and each patient was matched to a sex-matched control within the corresponding age category (Table S1).

For 12 of the 16 patients with sJIA sampled during active disease, samples from later inactive disease states were available and were also collected for comparative analysis. Clinical and laboratory data collected at each sampling occasion were retrieved from medical records and from the Swedish Paediatric Rheumatology Quality Register (Svenska Barnreumaregistret, Omda®) and are presented in Table S1.

### Proteomics assays

Plasma samples were subjected to proteomic profiling using the Olink Target 96 Neuro-exploratory Panel (Olink Bioscience, Sweden), high-throughput multiplex immunoassays based on proximity extension assay (PEA) technology. The Olink Target 96 Neuro-Exploratory panel measures 92 proteins involved in diverse neurobiological processes and disease mechanisms (https://olink.com/products/olink-target-96). We also mapped inflammatory proteins profile using the Inflammation panel which includes 92 proteins associated with inflammatory and immune response pathways.^22^ Protein abundance was quantified as log2-normalised protein expression (NPX) values. Proteins with NPX values below the limit of detection (LOD) in more than 80% of the samples were excluded. Differentially expressed proteins (DEPs) between groups were defined as those with a ΔNPX >1 (log2 fold change (FC) > 1) and an adjusted P value (P_adj_) < 0.05. P_adj_ were calculated using the Benjamini–Hochberg procedure to control the false discovery rate (FDR) at 5% within each comparison.

Principal component analysis (PCA) was performed on normalised protein expression data. The first three principal components were visualised in a three-dimensional PCA plot to assess sample clustering and overall variance structure. Hierarchical clustering analysis was performed using the ClustVis web tool (https://biit.cs.ut.ee/clustvis/) with Euclidean distance on z-score–normalised protein expression values from the Neuro Exploratory panel, and clustering was conducted using Ward's linkage.

### Induction of experimental arthritis and tissue collection

Female DBA/1 mice (8–10 weeks old) were obtained from Janvier Labs (France), and were housed at the Jagiellonian University in Krakow, Poland. Mice were randomly assigned to experimental groups using a random number generator prior to model induction. Collagen-induced arthritis (CIA) was induced as previously described.^23^ Female mice were chosen to avoid the potential confounding factor of spontaneous arthritis that can occur in male DBA/1 mice.^24^ Briefly, on day 0, 10-week-old mice (n = 6) were immunised with 100 μl of bovine type II collagen (CII; 100 μg, Chondrex, Inc., USA) emulsified in complete Freund's adjuvant (CFA; 50 μg *Mycobacterium tuberculosis*, Chondrex, Inc., USA) via intradermal injection at the base of the tail. 4 weeks later, a booster injection of 100 μl of CII (100 μg) emulsified in incomplete Freund's adjuvant (IFA, Chondrex, Inc., USA) was administered. Control mice (n = 6) received intradermal phosphate-buffered saline (PBS) injections at day 0 and 4 weeks later. Mice were monitored three times per week for signs of joint inflammation and scored using a 12-point scale, with each paw scored from 0 to 3 based on swelling and redness (0 = normal, 1 = mild, 2 = moderate, 3 = severe), giving a maximum possible score of 12 per mouse.^25^ The study was terminated on day 90.

Upon termination, mice were deeply anesthetised with isoflurane (Baxter, UK) and sacrificed by transcardial perfusion with cold PBS. Before perfusion, blood was collected via cardiac puncture and allowed to clot at room temperature for 30 min. Samples were then centrifuged at 2000 × *g* for 10 min at 4 °C, and serum was aliquoted and stored at −80 °C.

After perfusion, brain tissues were bisected along the midline. One half was used to isolate hippocampus and cortex for subsequent RNA and protein extraction. The other half of the brain tissues were immersed in 4% paraformaldehyde (PFA, Thermo Fisher, USA) in PBS for 24 h at room temperature. Then, tissues were cryoprotected in 20% sucrose in PBS for 48 h at 4 °C. Tissues were embedded in optimal cutting temperature compound (OCT, Leica Biosystems, USA) and then stored at −80 °C until sectioning. For the sectioning, tissues were cut into slices of 16 μm or 8 μm with the Cryostar NX7 (Thermo Fisher, USA). Slices were mounted on SuperFrost Plus adhesion glass slides (Thermo Fisher, USA) and stored at −80 °C for staining analysis.

### RNA and protein extraction from tissues

Hippocampal tissues were dissected and used for subsequent RNA and protein extraction. Tissues were homogenised with beads in 1 mL Qiazol lysis reagent (Qiagen, Germany) using the TissueLyser II (Qiagen, Germany). 200 μl of chloroform was added, followed by vigorous shaking for 15s. The mixture was incubated at room temperature for 3 min and centrifuged at 12,000 × *g* for 15 min at 4 °C to separate the phases. The aqueous phase was carefully transferred to a new tube for RNA precipitation. The interphase and organic phase were retained for protein extraction.

The RNeasy Lipid Tissue Kit (Qiagen, Germany) was used to isolate RNA from the tissues, according to manufacturer's protocol. The purity and concentration of RNA were measured by QIAxpert (Qiagen, Germany).

For protein extraction, 300 μl of 100% ethanol (Solveco AB, Sweden) was added to the interphase/organic phase, mixed thoroughly, and incubated for 3 min at room temperature, followed by centrifugation at 2000 × *g* for 5 min at 4 °C. The supernatant was transferred to a new tube, and proteins were precipitated by adding 1.5 mL of isopropanol (Solveco AB, Sweden). After incubation for 10 min at room temperature, the samples were centrifuged at 12,000 × *g* for 10 min at 4 °C. The resulting protein pellet was washed three times with 0.3 M guanidine hydrochloride (Sigma–Aldrich, USA) in 95% ethanol, followed by a final wash with 100% ethanol. The pellet was air-dried and resuspended in 1% sodium dodecyl sulphate (SDS) buffer for western blotting analysis.

### RNA sequencing and data analysis

RNA samples originating from 6 arthritic and 6 healthy mice were used for RNA sequencing analysis. Sequencing and library preparation were carried out at the Genomic Core Facility at the University of Bergen. Library preparation was performed using Illumina stranded mRNA preparation ligation kit with a total of 200 ng RNA. Illumina 10 bp unique dual indexes (UDI) were used and a total of eleven polymerase chain reaction (PCR) cycles were run. Briefly, mRNA was enriched from total RNA using poly(A) selection and converted into strand-specific libraries following the manufacturer's instructions. Sequencing was performed on an Illumina NovaSeq 6000 platform using a NovaSeq SP flowcell with a paired-end 2 × 100 bp setup. Raw data were processed using the standard Illumina pipeline to produce demultiplexed FASTQ files for downstream analysis. FASTQ files were subjected to quality control using FastQC. The results from all samples were aggregated using MultiQC (v1.11) to assess overall data quality. Clean reads were then aligned to the mouse genome (GRCm38.p5) using HISAT2 (v2.0.5) with the Gencode vM13 gene transfer format (GTF) file as the reference annotation. Gene-level counts were obtained using FeatureCounts (v1.5.2). The count matrix was generated and was used for downstream analysis.

Downstream analyses were performed using R software (v4.4.1). Briefly, genes with zero counts across all samples and non-protein coding genes were excluded from the data analysis. Normalisation was conducted using the median-of-ratios method implemented in DESeq2, with group (arthritic vs. healthy control mice) specified in the design formula (∼group). Differential expression was assessed using the Wald test. Raw p-values were adjusted for multiple testing using the Benjamini–Hochberg procedure as implemented in DESeq2 to control the FDR. Gene identifiers were further annotated with gene symbols and descriptions using Biomart (v2.61.3). To capture more potentially relevant biological signals, genes were considered differentially expressed when adjusted p-value (FDR) was <0.1 and the log2 FC was < −0.585 or > 0.585.

Three-dimensional PCA plots were generated using the plotly package (v4.10.3) in R, based on the complete RNA-seq expression matrix. Gene set enrichment analysis (GSEA) was performed using the mouse Molecular Signature Database (MSigDB),^26^ applying the Gene Ontology biological process (GO:BP) collection to a pre-ranked list of all genes from the RNA-seq dataset. Analysis was performed and results were visualised with GSEA software, version 4.3.3.

### Immunofluorescence staining

Before staining, the OCT-embedded tissue sections were dried at room temperature for 15 min. Antigen retrieval was performed using a sodium citrate buffer (sodium citrate 10 mM, 0.05% Tween-20, pH = 6.0), with one retrieval cycle of the 2100 antigen retriever (Aptum Biologics, UK). Sections were blocked in 0.3% Triton X-100 (Sigma–Aldrich, USA) and 5% serum (the type of serum depended on the antibodies used). Sections were incubated with the primary antibodies at 4 °C overnight. Next, sections were washed with PBS containing 0.02% Tween-20 before incubation with a secondary antibody for 1–2 h at room temperature. After another wash, sections were incubated with 0.1% Sudan Black B (Sigma–Aldrich, USA) in 70% ethanol for 20 min and room temperature. Next, slides were mounted using ProLong™ Gold Antifade Mountant with DNA Stain DAPI (Thermo Fisher, USA). Images were captured with the Zeiss LSM-880 confocal microscope. Fluorescence intensity quantification was performed using ImageJ (version 1.54). The details of primary and secondary antibodies are listed in Table S2.

### Image analysis

Microglial morphology was analysed using the skeleton analysis plugins in Fiji/ImageJ software (NIH, USA). Briefly, confocal z-stack images of IBA1-stained microglia were first converted to 8-bit grayscale, and brightness/contrast was adjusted to optimise visualisation. Images were then processed with a Gaussian blur filter to enhance continuous structures, followed by thresholding to generate binary masks that captured the main cellular processes. In cases of background noise, the Despeckle function was applied to improve image clarity. The resulting binary images were converted into skeletons using the Skeletonise plugin. Branching parameters were quantified using the Analyse Skeleton (2D/3D) tool.

For quantification of fluorescence intensity, fluorescence images were processed and quantified using Fiji/ImageJ software (NIH, USA). The mean fluorescence intensity for each channel was measured within the regions of interest (ROIs) using the measure function in Fiji. A consistent automatic thresholding method was applied to segment the signal from the background. Regions outside the signal, including scale bars and artifacts, were excluded. Mean fluorescence intensity was measured within the threshold-defined area. All images were acquired under identical microscope settings to ensure comparability.

### Western blotting

Protein Assay Dye Reagent Concentrate (Bio-Rad, USA) was used to measure the concentration of extracted proteins following the manufacturer's instructions. For each sample, 15ug protein was mixed with 4 × SDS loading buffer (Bio-Rad, USA), heated at 95 °C for 5 min, and separated by 4–20% Mini Protean TGX gels (Bio-Rad, USA). Sample transfer to 0.2 μm nitrocellulose membranes (Amersham™ Protran™, Cytiva, Germany) using the Fisherbrand™ Semi-Dry Blotter (Thermo Fisher Scientific, UK) was performed according to the manufacturer's protocol. Membranes were then blocked with 5% nonfat milk (Cell signalling technology, USA) Tris-buffered saline with Tween-20 (TBST) for 1 h at room temperature, followed by overnight incubation at 4 °C with primary antibodies. After three washes with TBST, membranes were incubated with species-appropriate horseradish peroxidase (HRP)-conjugated secondary antibodies for 1 h at room temperature. The details of primary and secondary antibodies are listed in Table S2.

Signal detection was performed using Clarity™ Western ECL substrate (Bio-Rad, USA), and membranes were imaged with ChemiDoc™ MP imaging system (Bio-Rad, USA). Band intensities were quantified using ImageJ software (version 1.54) and normalised to loading control proteins.

### Enzyme-linked immunosorbent assays (ELISA)

Mouse serum samples and mouse microglia cell culture supernatants were used to measure HMOX2 with a commercial HMOX2 kit (MyBiosourse, USA) according to manufacturer's instructions. Mouse serum samples were also used to measure IL-6 and IL-18 level using IL-6 and IL-18 mouse Elisa kits (R&D systems, USA). Optical density (OD) values were measured using the SpectraMax microplate reader (Molecular Devices, USA).

### Cell culture and measurement of ROS production via 2′,7′-dichlorodihydrofluorescein diacetate (DCFH-DA) assay

The mouse microglia cell line SimA9 (RRID: CVCL_5I31) was cultured in DMEM/F12 GlutaMAX™ culture media (Gibco, UK), supplemented with 10% foetal bovine serum (FBS, Sigma–Aldrich, USA), 5% horse serum (Sigma–Aldrich, USA), and 1% penicillin–streptomycin (Gibco, UK). The cell line was verified as free from contamination through mycoplasma testing. Cells were maintained either in 6-well plates (1.5 × 10^5^/well) or in 8-chamber polystyrene tissue culture–treated glass slides (1 × 10^4^/chamber). Prior to treatment with H_2_O_2_, IL-6, or IL-18, cells were preconditioned in Opti-MEM (Gibco, UK) overnight. They were then treated with 100 μM hydrogen peroxide (H_2_O_2_, Merck, Germany), 20 ng/mL IL-6 (R&D systems, USA), or 50 ng/mL IL-18 (MedChemExpress, USA) in Opti-MEM for 4, 8, or 24 h.

After treatment, culture supernatants from the 6-well plates were collected for subsequent HMOX2 ELISA measurements, and proteins from cells were extracted for western blotting. For ROS detection, cells seeded in 8-chamber slides were washed with PBS and incubated with 10 μM DCFH-DA (Sigma–Aldrich, USA) for 30 min at 37 °C. The probe solution was then removed, cells were washed with PBS, and fluorescence signal was acquired using a Zeiss LSM 880 confocal microscope. Fluorescence intensity was quantified using ImageJ software (version 1.54).

### Ethics

Samples from patients with JIA and from healthy children were collected in accordance with the Declaration of Helsinki. Ethical approval was obtained from the North Ethical Committee in Stockholm, Sweden (Dnr 2009-1139-30-4 and Dnr 2010-165-31-2 for JIA patient samples; Dnr 03–067 for the samples from HCs). All animal procedures were performed in compliance with protocols approved by the First Regional Ethical Committee for Animal Experiments in Kraków, Poland (approval number: 329/2022). Written informed consent was obtained from all participants or, in the case of participants unable to provide consent themselves, from their legal guardians or parents.

### Statistics

For human plasma proteomics data analyses, multiple paired t-test was performed to compare sJIA samples (n = 16) with HCs (n =16), followed by Benjamini–Hochberg false discovery rate (FDR) correction. To compare categorical variables such as sex ratio, and treatment distribution between the two sJIA clusters, defined based on the neurobiological protein profile, Fisher's exact test was used. Age and disease duration between clusters were evaluated with the Mann–Whitney U test. Spearman correlation analyses with the Benjamini–Hochberg adjustment were used to determine correlations between target proteins and selected clinical parameters. For longitudinal comparisons, multiple paired t-tests with Holm–Bonferroni adjustment were applied to assess changes between the active and inactive phases in paired samples from patients with sJIA (n = 12) and HCs (n = 12). Mouse Experimental data were analysed with an unpaired t-test, and Spearman correlation analysis was performed to identify the correlations between HMOX2/IL-6/IL-18 levels in mouse serum samples and CNS changes. RNA sequence data was analysed as described in the RNA sequencing and data analysis section. Statistical analysis was performed using GraphPad Prism 9.0.0 (GraphPad Software, USA) or R software (v4.4.1).

For Olink profiling, proteins with |log2 FC| > 1 and an FDR-adjusted p-value <0.05 were considered statistically significant. For the RNA-seq analysis, significance was defined as FDR-adjusted p-value <0.1 and |log2 FC| > 0.585. For single predefined comparison without multiple testing in patients and mice, a p-value <0.05 was considered statistically significant. For analyses involving multiple comparisons within the sJIA cohort, adjusted p-value <0.05 were considered significant.

### Role of funders

The funders had no role in the study design, data collection and analyses, results interpretation, or writing of the manuscript.

## Results

### Patients with active sJIA exhibit an altered neurobiological protein profile in plasma compared with healthy individuals

**Fig. 1:**
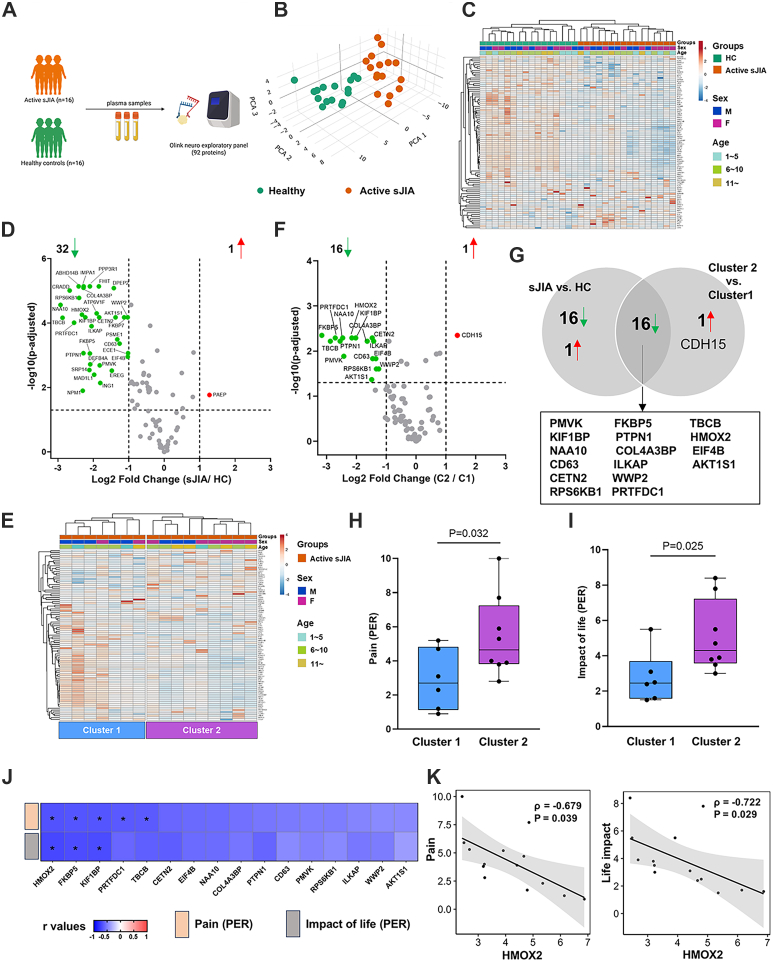
**Patients with sJIA exhibit an altered neurobiological protein profile, which is associated with higher pain and greater life impact. (A)** Schematic diagram of a cross-sectional study profiling neurobiological related proteins, including 16 active sJIA, and 16 age- and sex-matched HCs. **(B)** PCA plot of neuro-related plasma proteins showing separation between patients with sJIA and HCs. Each point represents one subject (n = 32; 16 active sJIA, and 16 HCs). **(C)** Heatmap of protein expression in sJIA and HCs, with hierarchical clustering revealing separation between sJIA and HCs. **(D)** Volcano plot of DEPs in active sJIA vs. HCs. Dashed lines indicate significance thresholds. **(E)** Unsupervised hierarchical clustering analysis grouping patients with active sJIA into two clusters based on their neurobiological protein expression patterns. **(F)** Volcano plot of DEPs in Cluster 2 vs. Cluster 1. Dashed lines indicate significance thresholds. **(G)** Venn diagram showing overlapping DEPs between active sJIA vs. HCs and Cluster 2 vs. Cluster 1. **(H)** Box plot comparing pain scores between patients with active sJIA in Cluster 1 and Cluster 2. One patient in each cluster is missing pain score data. **(I)** Box plot comparing life impact scores between patients with active sJIA in Cluster 1 and Cluster 2. One patient in each cluster is missing life impact score data. **(J)** Heatmap of correlations between DEPs and pain/life impact in patients with active sJIA. **(K)** Correlation analysis showing HMOX2 as the strongest negatively correlated protein with pain and life impact in patients with active sJIA, illustrated by scatter plot. Statistics: (D) paired t-test with Benjamini–Hochberg correction for multiple comparisons; (F) unpaired t-test with Benjamini–Hochberg correction for multiple comparisons; (H, I) unpaired t-test; (J, K) Spearman correlation analysis with Benjamini–Hochberg correction. ∗: *P* < 0.05.

We collected plasma samples from patients with active sJIA and age- and sex-matched HCs, and profiled neurobiologically related proteins using the Olink Neuro Exploratory panel (Fig. 1A). Three-dimensional PCA revealed a clear separation between HCs and patients with sJIA (Fig. 1B). Hierarchical cluster analysis also demonstrated a distinct separation between the two groups and the relative abundance of each protein across individuals was visualised in a heatmap (Fig. 1C). We then identified the DEPs using a paired t-test, with a threshold of |log2 FC| > 1 and Benjamini–Hochberg adjusted p-value <0.05. Overall, 32 proteins were significantly downregulated in patients compared to HCs, while one protein, progestagen associated endometrial protein (PAEP) was upregulated (Fig. 1D, Table S3).

### The altered neurobiological protein profile links to higher pain and life impact in patients with sJIA and associated with disease duration

To further explore neurobiological proteomic alterations in sJIA, we performed unsupervised hierarchical clustering based on proteomic profiles from patients with active sJIA. This analysis revealed two distinct clusters in sJIA, comprising 7 patients in Cluster 1 and 9 patients in Cluster 2 (Fig. 1E). Interestingly, the differences in protein expression between these two clusters mirrored those observed when comparing sJIA with HCs. Specifically, in cluster 2, a total of 16 proteins were downregulated compared with cluster 1, and these proteins also showed decreased levels in the overall comparison between sJIA and HCs. One protein, cadherin-15 (CDH15) was upregulated in cluster 2 as compared with cluster 1 (Fig. 1F and G, Table S4).

To assess the clinical significance of these proteomic changes, we compared clinical parameters reflecting neurobiological well-being between the two clusters. As psychometric data was unavailable for the sJIA cohort, patient-reported pain and life impact scores were used as surrogate indicators of neurological status. Patients in Cluster 2 reported higher pain (*P* = 0.032, unpaired t test) and life impact scores (*P* = 0.025, unpaired t test) compared to those in Cluster 1 (Fig. 1H and I). We then examined the associations between 16 downregulated proteins (from Fig. 1G) and patient-reported pain and life impact scores in sJIA. Spearman correlation analysis identified 5 proteins negatively associated with pain and 3 proteins negatively associated with life impact in patients with active sJIA (Fig. 1J). Among these, HMOX2 showed the strongest negative correlation (lowest adjusted-p value) with both pain (Spearman r = −0.679, *P*_*adj*_ = 0.039) and life impact (Spearman r = −0.722, *P*_*adj*_ = 0.029) (Fig. 1K–Table S5–S6). These results indicate that the altered neurobiological profile may reflect underlying neurofunctional impairments and suggest mechanisms potentially affecting psychiatric well-being.

To determine the factors influencing clustering based on neurobiological profiles, we compared the demographics and disease characteristics of patients in the two clusters, including sex, age at onset, age at sampling, disease duration, disease activity, C-reactive protein (CRP) measurements, autoantibodies, and treatment. Notably, patients in Cluster 2 had a substantially longer disease duration (Table S7) (*P* = 0.003, Mann–Whitney U test). No other significant differences were observed between the two clusters, and treatment regimens did not differ, suggesting that these factors were unlikely to account for the observed clustering (Figure S1A, Table S7).

### Altered neurobiological protein profiles persist during the inactive phase of sJIA

**Fig. 2:**
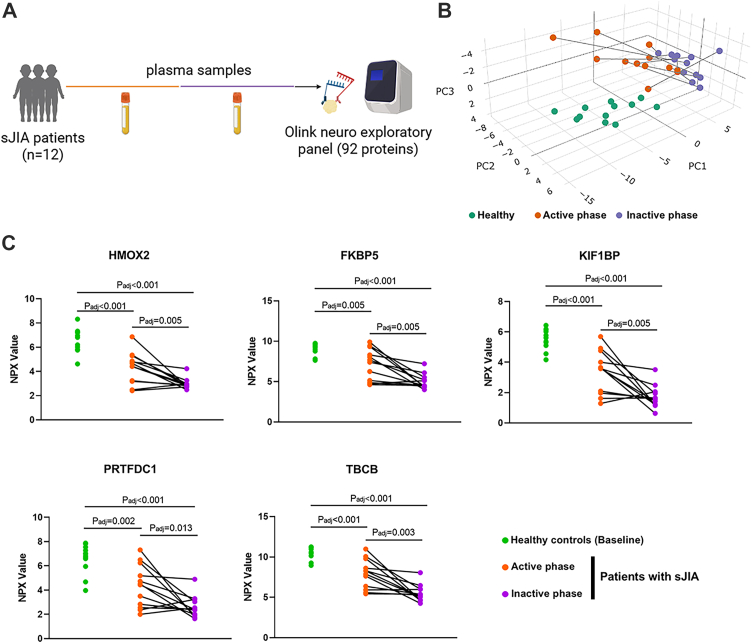
**Paired comparison of neurobiological protein profile changes in 12 patients with sJIA across active and inactive phases, with 12 age- and sex-matched HCs. (A)** Schematic diagram of a paired study profiling neurobiological related proteins, including 12 patients with sJIA with samples from both active and inactive phases, and 12 age- and sex-matched HCs. **(B)** PCA plot of neurobiological related plasma proteins showing the distribution of patients with sJIA (active vs inactive) and HC. Each point represents one subject, with lines connecting paired active and inactive samples from the same patient (n = 36; 12 active sJIA, 12 inactive sJIA, and 12 HCs). **(C)** Line plot showing longitudinal changes in proteins identified as related to clinical parameters, from active to inactive sJIA, using age- and sex-matched healthy individuals as the baseline. Statistics: (C) Multiple paired t-tests with Holm–Bonferroni adjustment.

Among the 16 patients with active sJIA, 12 also had plasma samples collected later during the inactive phase. We compared neurobiological profiles between paired samples from these patients, with age- and sex-matched HCs serving as a baseline (Fig. 2A). PCA revealed that inactive sJIA samples were, unexpectedly, more distinct from HCs than active sJIA samples (Fig. 2B). Interestingly, all proteins previously identified as negatively correlated with pain and life impact were even lower in the inactive phase compared with the active phase (Fig. 2C). These findings suggest that even after clinical remission of active sJIA, neurobiological alterations may continue to progress.

### Neuroinflammation and impaired neurogenesis in mice with chronic arthritis

**Fig. 3:**
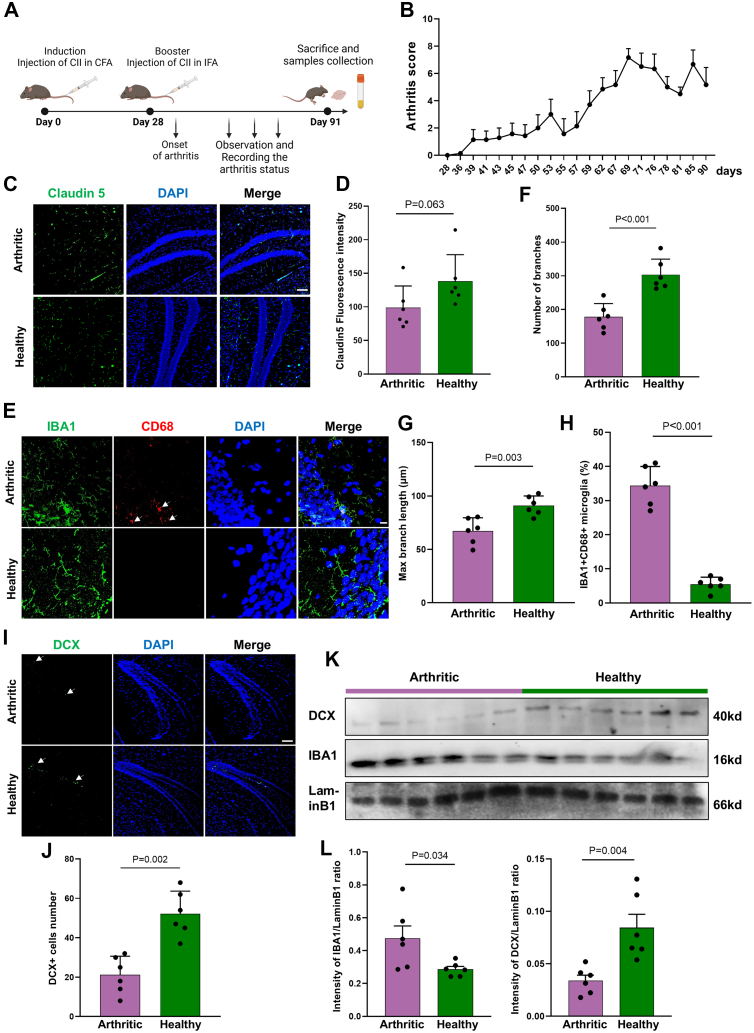
**Chronic arthritic mice exhibit neuroinflammation and impaired neurogenesis in the hippocampus. (A)** Schematic diagram of the chronic arthritis mouse model (collagen-induced arthritis, CIA). **(B)** Line plot showing changes in arthritis scores of immunised mice over the disease course. **(C)** Representative images of immunofluorescence (IF) staining for Claudin-5 in the hippocampus of arthritic (n = 6) and healthy (n = 6) mice. Scale Bar: 100 μm. **(D)** Box plots quantifying Claudin-5 fluorescence intensity by IF in arthritic and healthy mice. **(E)** Representative images of IF staining for microglial markers (IBA1 and CD68) in the hippocampus of arthritic (n = 6) and healthy (n = 6) mice. Scale Bar: 10 μm. **(F**–**G)** Box plots quantifying microglial morphology (branch number and branch length) in arthritic and healthy mice. **(H)** Box plots showing the number of activated microglial cells (IBA1^+^ CD68^+^) by IF in arthritic and healthy mice. **(I)** Representative images of IF staining for DCX in the hippocampus of arthritic (n = 6) and healthy (n = 6) mice. Scale Bar: 100 μm. **(J)** Box plots quantifying the number of DCX + neurons by IF in arthritic and healthy mice. **(K**–**L)** Western blotting results showing downregulation of DCX and upregulation of IBA1 in arthritic hippocampus compared with controls. Statistics: (D, F, G, H, J, L) unpaired t-test.

As no animal model currently fully recapitulates the complex features of sJIA, we employed the well-described CIA model and maintained mice for 3 months to mimic features of chronic autoimmune arthritis and to further investigate the link between arthritis and neurobiological alterations (Fig. 3A). The arthritis score peaked on day 69 and subsequently fluctuated, resembling the flare and remission pattern observed in patients (Fig. 3B). Arthritis parameters were quantified for each mouse (Figure S2A).

We focused our analysis on the hippocampus and cortex, regions known to be critical for cognition and mood regulation.^27^ In the hippocampus, we investigated Claudin-5 expression, a tight junction protein critical for maintaining BBB integrity.^28^ We observed a slight decrease in arthritic mice compared with healthy mice, but the change was not statistically significant (Fig. 3C-D) (*P* = 0.063, unpaired t test). In contrast, microglia in arthritic mice exhibited a pronounced amoeboid morphology, characterised by fewer and shorter branches (Fig. 3E–G) (number of branches: *P* < 0.001, branch length: *P* = 0.003, unpaired t tests). IBA1^+^CD68^+^ microglia cells were significantly increased in the hippocampus of arthritic mice (*P* < 0.001, unpaired t test), both features being associated with microglial activation and indicative of neuroinflammation (Fig. 3E and H). Furthermore, neurogenesis appeared impaired, as evidenced by a reduction in DCX^+^ neuronal cells in the hippocampus of arthritic mice (Fig. 3I and J) (*P* = 0.002, unpaired t test). These observations were further validated by Western blot analysis (Fig. 3K and L).

In contrast, no significant changes were observed in the cortex between arthritic and healthy mice. Claudin-5 expression in the cortex showed no notable difference (Figures S2B–S2C). Similarly, microglial activation, assessed by IBA1 and CD68 staining, did not show significant alterations (Figures S2D–S2F).

### Mitochondrial dysfunction and oxidative stress are important contributors to hippocampal alterations in experimental chronic arthritis

To further elucidate the molecular mechanisms underlying neuroinflammation, BBB integrity and decreased neurogenesis, as suggested by immunofluorescence analysis of the hippocampus of arthritic mice, we isolated hippocampal tissues from both arthritic and healthy control mice for transcriptomic profiling using RNA sequencing. Sequence quality was assessed by FastQC and MultiQC analysis, including duplicate read analysis, per-base and per-sequence quality metrics, and per-sequence GC content (Fig S3A–S3D).

**Fig. 4:**
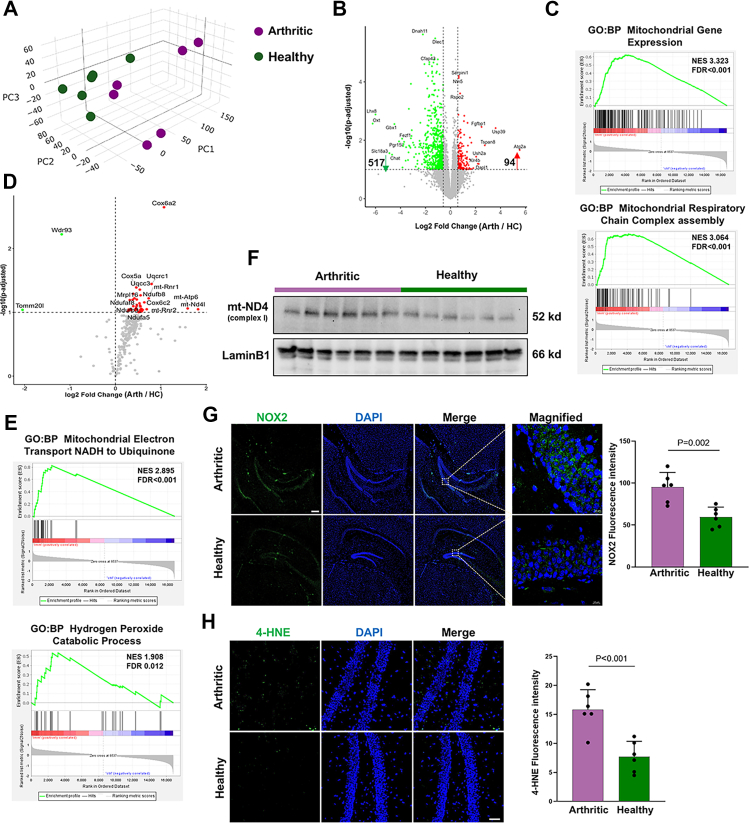
**RNA-seq analysis identifies mitochondrial dysfunction and oxidative stress associated with hippocampal neuroinflammation in arthritic mice. (A)** PCA plot of RNA-seq data showing separation between arthritic (n = 6) and healthy (n = 6) mice. **(B)** Volcano plot displaying 94 upregulated and 517 downregulated genes in the hippocampus of arthritic mice compared with healthy mice. Dashed lines indicate significance thresholds. **(C)** GSEA plots showing activation of pathways related to mitochondria and the mitochondrial respiratory chain in arthritic mice. **(D)** Volcano plot showing mitochondrial-related DEGs in the hippocampus of arthritic mice compared with controls. Dashed lines indicate significance thresholds. **(E)** GSEA plots identifying activation of pathways involving mitochondrial complex I and the hydrogen peroxide catabolic process in arthritic mice. **(F)** Western blot results showing upregulation of mt–ND4 in arthritic hippocampus compared with controls. **(G)** Representative images of IF staining for NOX2 in the hippocampus of arthritic (n = 6) and healthy (n = 6) mice, with corresponding box plot quantifying NOX2 fluorescence intensity. Scale Bar: 200 μm. **(H)** Representative images of IF staining for 4-HNE in the hippocampus of arthritic (n = 6) and healthy (n = 6) mice, with corresponding box plot quantifying 4-HNE fluorescence intensity. Scale Bar: 50 μm. Statistics: (B, D) RNA-seq counts normalised and differential expression calculated using the DESeq2 package; (G, H) unpaired t-test.

PCA plot of the hippocampal transcriptomes revealed a separation between arthritic and control groups (Fig. 4A), indicating substantial transcriptional changes in the hippocampus during arthritis. Differential gene expression analysis identified 94 upregulated and 517 downregulated genes in the hippocampus of arthritic mice as compared with controls (Fig. 4B). We performed gene set enrichment analysis to explore dysregulated pathways. Pathways related to neurogenesis (e.g., Positive regulation of neural precursor cell proliferation, and forebrain neuron development), BBB integrity (e.g., Establishment of endothelial barrier, and extracellular structure organisation) were downregulated in arthritic mice (Figure S4A), supporting the observed hippocampal dysfunction and aligning with our histological findings.

Interestingly, GSEA revealed that the predominantly upregulated pathways in the hippocampus of arthritic mice were related to mitochondrial function and the mitochondrial respiratory chain (Fig. 4C, Table S8). Based on this observation, we hypothesised that mitochondrial dysfunction may contribute to neuroinflammation in the hippocampus. To explore this possibility, we curated a list of mitochondria-related genes (based on GO:0005739 dataset) and identified differentially expressed genes (DEGs, P_adj_ < 0.1). In total, 40 mitochondria-associated genes were upregulated, while only 2 were downregulated in the hippocampus of arthritic mice (Fig. 4D). Notably, many of these DEGs were components of mitochondrial complex I (e.g., mt–ND4L, Ndufaf8, Ndufb3, Ndufa5, and Ndufb8). Mitochondrial complex I is an important site of ROS production and has previously been reported to exhibit increased activity in microglia, thereby sustaining neuroinflammation in chronic inflammatory disorders.^29^^,^^30^ Our GSEA results also demonstrated that pathways related to mitochondrial complex I and oxidative stress were significantly enriched in the hippocampus of arthritic mice (Fig. 4E). Therefore, we investigated mitochondrial complex I activity and oxidative stress in the hippocampus of arthritic and healthy mice. Immunofluorescence staining showed abundant complex I expression in both groups, with no significant differences detected (Figure S4B) (*P* = 0.377, unpaired t test), possibly due to high baseline expression in hippocampal tissue. However, Western blot analysis revealed increased mt–ND4 protein levels in the hippocampus of arthritic mice (Fig. 4F, Figure S4C) (*P* = 0.007, unpaired t test). Moreover, NADPH oxidase 2 (NOX2), a key contributor to ROS production, was markedly upregulated in the hippocampus of arthritic mice (Fig. 4G) (*P* = 0.002, unpaired t test). Additionally, immunostaining for 4-hydroxynonenal (4-HNE), a lipid peroxidation marker of oxidative stress, also revealed increased levels in the arthritic group (Fig. 4H) (*P* < 0.001, unpaired t test). These findings indicate elevated oxidative stress in the hippocampus of arthritic mice and involved in neuroinflammation during chronic arthritis.

### HMOX2 is downregulated in arthritic mice, and serum HMOX2 levels are negatively correlated with hippocampal neuroinflammation

In the analyses of sJIA, HMOX2 emerged as a protein of particular interest. HMOX2 has a well-established role in oxidative stress regulation and our RNA-seq data emphasised the involvement of oxidative stress in the hippocampus of arthritic mice. More importantly, its plasma expression was markedly downregulated in patients with sJIA and demonstrated the strongest negative correlation (Fig. 1J and K) with clinical measures of pain and life impact in patients with active sJIA. Given its tissue-enriched expression in the brain and testis,^31^ these findings prompted us to further investigate a potential biomarker role of HMOX2 for neuroinflammation associated with chronic arthritis.

**Fig. 5:**
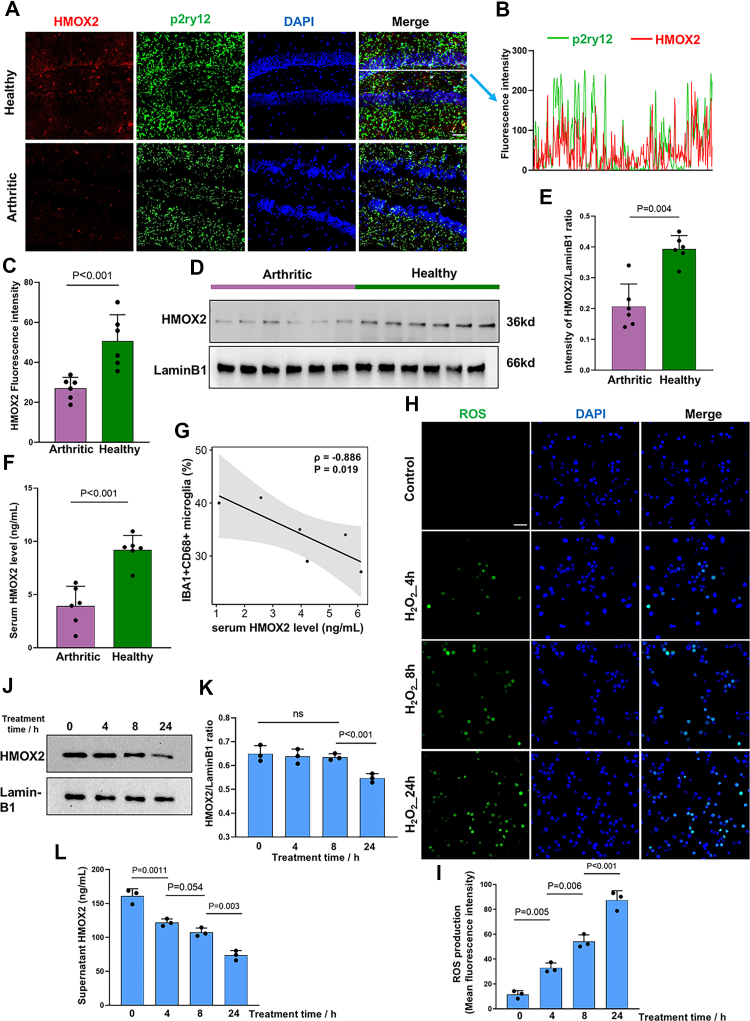
**HMOX2 is decreased in the hippocampus and serum of arthritic mice, and its downregulation is associated with microglial oxidative stress. (A)** Representative images of IF staining for HMOX2 and the homoeostatic microglial marker P2ry12 in the hippocampus of arthritic (n = 6) and healthy (n = 6) mice. Scale Bar: 50 μm. **(B)** Intensity profiles of HMOX2 (red) and P2ry12 (green) along the line indicated in Fig. 5A in hippocampal tissue. **(C)** Box plots quantifying HMOX2 fluorescence intensity by IF in arthritic and healthy mice. **(D**–**E)** Western blot results showing decreased HMOX2 in the hippocampus of arthritic mice compared with controls. **(F)** ELISA results showing reduced serum HMOX2 levels in arthritic mice compared with controls. **(G)** Correlation analysis showing a negative correlation between serum HMOX2 and microglial activation in arthritic mice. **(H–I)** Representative images of DCFH-DA staining showing ROS levels in SimA9 cells treated with H_2_O_2_ for 4, 8, and 24 h, with corresponding quantification of fluorescence intensity. Scale bar: 50 μm. **(J**–**K)** Western blot results showing intracellular HMOX2 changes in SimA9 cells treated with H_2_O_2_ for 4, 8, and 24 h, with corresponding quantification of grayscale intensity. **(L)** ELISA results showing downregulation of extracellular HMOX2 in SimA9 cells following H_2_O_2_ treatment. Statistics: (C, E, F) unpaired t-test; (G) Spearman correlation analysis; (I, K, L) three independent experiments, one-way ANOVA followed by Tukey's multiple comparisons test to assess differences among groups.

We first examined HMOX2 expression in hippocampal tissue. Immunofluorescence staining revealed that HMOX2 was abundantly expressed in hippocampus of healthy mice, with prominent localisation in homoeostatic microglia, as demonstrated by co-localisation with P2Y purinergic receptor 12 (P2ry12), a marker of homoeostatic microglia^32^ (Fig. 5A and B). In contrast, HMOX2 expression was significantly decreased in the hippocampus of arthritic mice (Fig. 5C–E) (IF quantification: *P* < 0.001, Western blot quantification: *P* = 0.004, unpaired t tests). Consistently, serum levels of HMOX2 were also markedly reduced in arthritic mice (Fig. 5F). Importantly, serum HMOX2 levels were negatively correlated with microglial activation in the hippocampus, as indicated by the frequency of IBA1^+^CD68^+^ microglia (Fig. 5G) (Spearman r = −0.886, *P* = 0.019, spearman correlation). Given that HMOX2 also exhibited a strong negative correlation with pain severity and life impact scores in patients with sJIA (Fig. 1J and K), peripheral HMOX2 may serve as a promising biomarker reflecting neurobiological alterations in the brain during chronic arthritis.

Based on the evidence of oxidative stress in the hippocampus of arthritic mice and reduced HMOX2 expression observed in both murine and clinical samples, we sought to explore the mechanistic link between peripheral HMOX2 and neuroinflammation. To this end, we conducted *in vitro* experiments using SimA9, a spontaneously immortalised mouse microglial cell line^33^ selected due to our findings that HMOX2 is expressed in homoeostatic microglia in mouse hippocampus. To mimic oxidative stress conditions in microglia, we treated SimA9 cells with H_2_O_2_. ROS production was significantly elevated as early as 4 h post-treatment, confirming the induction of oxidative stress (Fig. 5H and I). Western blot analysis revealed that intracellular HMOX2 protein levels remained stable up to 8 h but was decreased at 24 h after H_2_O_2_ treatment (Fig. 5J and K) (8 h vs 24 h: *P* < 0.001, one-way ANOVA followed by Tukey's multiple comparisons test). In contrast, ELISA results indicated a steady decline in extracellular HMOX2 levels during the whole period (Fig. 5L). These findings suggest that oxidative stress reduces the release of HMOX2 from microglial cells. The reduction in circulating HMOX2 may be linked to systemic oxidative stress and indicate the development of hippocampal neuroinflammation.

### IL-6 and IL-18 are key cytokines contributing to neuroinflammation during chronic arthritis

Previous studies have suggested that peripheral inflammatory cytokines may contribute to CNS neuroinflammation under pathological conditions such as autoimmune diseases, surgical trauma, and chronic infections.^34^, ^35^, ^36^ To identify key cytokines potentially involved in neurobiological alterations during chronic arthritis, we profiled the inflammatory mediator landscape in the same sJIA samples using the Olink inflammation panel and performed a cross-panel correlation analysis between the two Olink datasets to evaluate the overlap and consistency of protein expression changes.

**Fig. 6:**
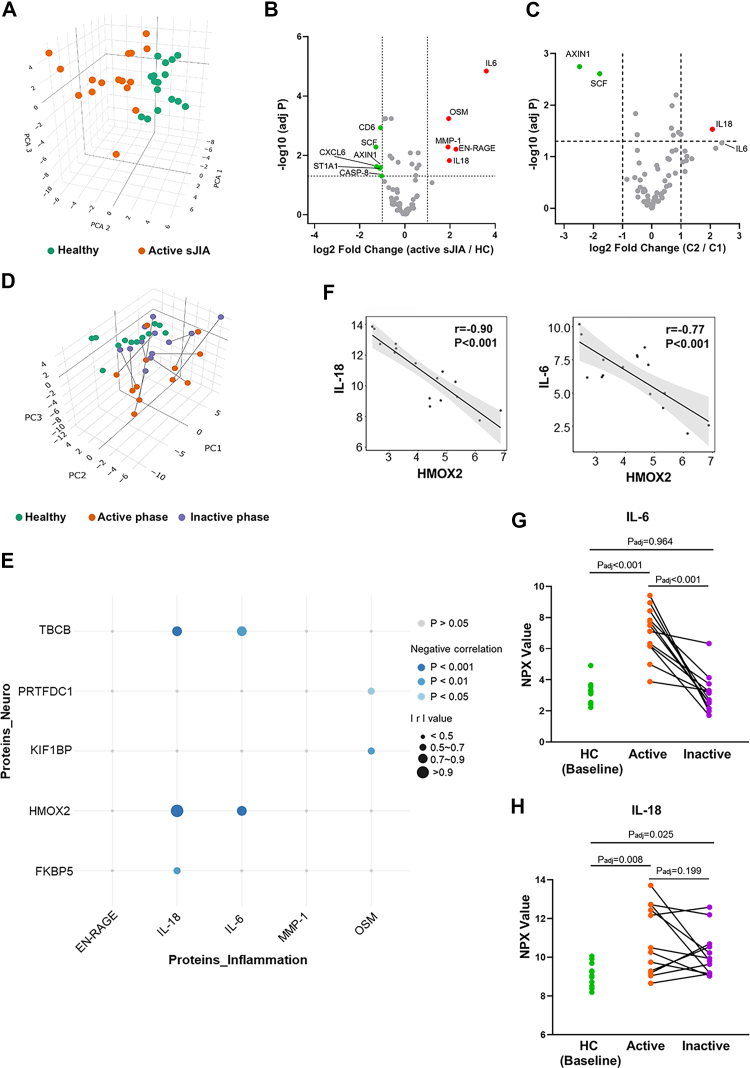
**Inflammatory profiles of patients with sJIA and neuro–inflammation co-analysis reveal correlations between HMOX2 and IL-6/IL-18. (A)** PCA plot of inflammation-related plasma proteins showing separation between patients with active sJIA and HCs. Each point represents one subject (n = 32; 16 active sJIA and 16 HCs). **(B)** Volcano plot of DEPs in active sJIA vs. HC. Dashed lines indicate significance thresholds. **(C)** Volcano plot of DEPs in Cluster 2 active sJIA vs. Cluster 1 active sJIA. Dashed lines indicate significance thresholds. **(D)** PCA plot of inflammation related plasma proteins showing the distribution of patients with sJIA (active vs inactive) and HCs. Each point represents one subject, with lines connecting paired active and inactive samples from the same patient (n = 36; 12 active sJIA, 12 inactive sJIA, and 12 HCs). **(E)** Bubble plot showing correlations between upregulated proteins from the inflammation panel and DEPs associated with clinical pain and life impact from the neuro panel in patients with active sJIA. **(F)** Correlation analysis showing significant negative correlations between HMOX2 and IL-6/IL-18. **(G**–**H)** Line plot showing longitudinal changes of IL-6 and IL-18 from healthy to active to inactive sJIA. Statistics: (B) paired t-test with Benjamini–Hochberg correction for multiple comparisons; (C) unpaired t-test with Benjamini–Hochberg correction for multiple comparisons; (E, F) Pearson correlation analysis; (G, H) Multiple paired t-tests with Holm–Bonferroni adjustment.

PCA plot demonstrated that patients with active sJIA exhibited a distinct inflammatory signature, clearly separated from HCs (Fig. 6A). Among the differentially expressed proteins, IL-6, IL-18, OSM, EN-RAGE, and MMP-1 were significantly elevated in active sJIA compared with HCs. Conversely, SCF, CD6, AXIN1, ST1A1, CXCL6, and CASP-8 were significantly decreased (Fig. 6B, Table S9, paired t test with Benjamini–Hochberg adjustment). We then compared the inflammation profiles between two sJIA clusters which were defined based on neurobiological protein profiles. IL-18 was significantly increased in cluster 2 (*P*_adj_ = 0.029, unpaired t test with Benjamini–Hochberg adjustment), while AXIN1 and SCF were decreased (AXIN1: *P*_adj_ = 0.002, SCF: *P*_adj_ = 0.002, unpaired t test with Benjamini–Hochberg adjustment). We also noted a trend toward increased IL-6 levels in cluster 2 (*P*_adj_ = 0.054, unpaired t test with Benjamini–Hochberg adjustment) (Fig. 6C, Table S10). In addition, paired comparisons revealed that, unlike the continued progression of the neurobiological profile in the inactive phase, the inflammation profiles of patients with sJIA partially returned to baseline when they were in an inactive phase (Fig. 6D).

To further explore the potential interplay between inflammatory proteins and neurobiological alterations, we performed correlation analyses between upregulated inflammatory proteins and neurobiological related DEPs associated with clinical measures of pain and life impact in all patients with active sJIA. IL-6, and IL-18 exhibited significant negative correlations with HMOX2 (Fig. 6E and F). We also investigated the IL-6 and IL-18 changes between active and inactive phase using the paired samples. We found that IL-6 increased significantly in the active phase but returned to baseline in the inactive phase, while higher level of IL-18 could still be observed in inactive samples (Fig. 6G and H) (inactive sJIA vs HCs: *P*_adj_ = 0.025, multiple paired t tests with Holm–Bonferroni adjustment). Given the observed elevation of IL-6 and IL-18 in patients with active sJIA and their negative association with HMOX2, we speculated that IL-6 and IL-18 may play important roles in neuroinflammation during chronic arthritis.

**Fig. 7:**
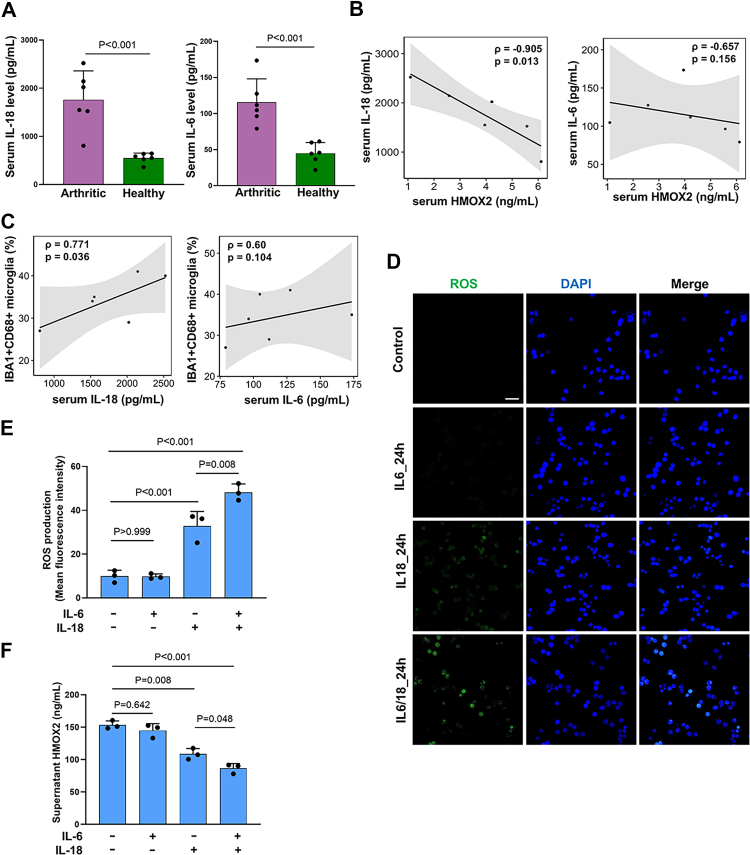
**IL-6 and IL-18 are elevated in arthritic mice and synergistically trigger oxidative stress in microglia. (A)** Box plots showing increased serum IL-6 and IL-18 levels in arthritic mice. **(B)** Correlation analysis showing a negative correlation between serum IL-18 and HMOX2, whereas IL-6 showed no significant correlation in arthritic mice. **(C)** Scatter plots showing the correlations between serum IL-18/IL-6 and microglial activation in arthritic mice. **(D**–**E)** Representative images of DCFH-DA staining showing ROS levels in SimA9 cells treated with IL-6, IL-18, or both for 24 h, with corresponding quantification of fluorescence intensity. Scale Bar: 50 μm. **(F)** ELISA results showing downregulation of extracellular HMOX2 in SimA9 cells following IL-18 treatment or IL-6/IL-18 co-treatment. Statistics: (A) unpaired t-test; (B, C) Spearman correlation analysis; (E, F) three independent experiments, one-way ANOVA followed by Tukey's multiple comparisons test to assess differences among groups.

To experimentally validate this hypothesis, we first assessed IL-6 and IL-18 levels in serum samples from mice with CIA. Consistent with our clinical observations, both IL-6 and IL-18 were significantly elevated in arthritic mice compared with healthy control mice (Fig. 7A) (IL-6 & IL-18: *P* < 0.001, unpaired t tests). Serum levels of IL-18 were negatively correlated with HMOX2 levels (Spearman r = −0.905, *P* = 0.013, spearman correlation), while the correlation with IL-6 did not reach significance in arthritic mice (Spearman r = −0.657, *P* = 0.156, spearman correlation) (Fig. 7B). But consistency with IL-18 and IL-6 patterns observed in patients with sJIA were evident. We next examined the relationship between IL-6/IL-18 and microglial activation. Serum IL-6 levels did not show a positive correlation with microglial activation (Spearman r = 0.600, *P* = 0.104, spearman correlation), while serum IL-18 levels exhibited a strong positive correlation with microglial activation (Spearman r = 0.771, *P* = 0.036, spearman correlation) (Fig. 7C), suggesting a pronounced role for IL-18 in driving microglial inflammatory responses.

**Fig. 8:**
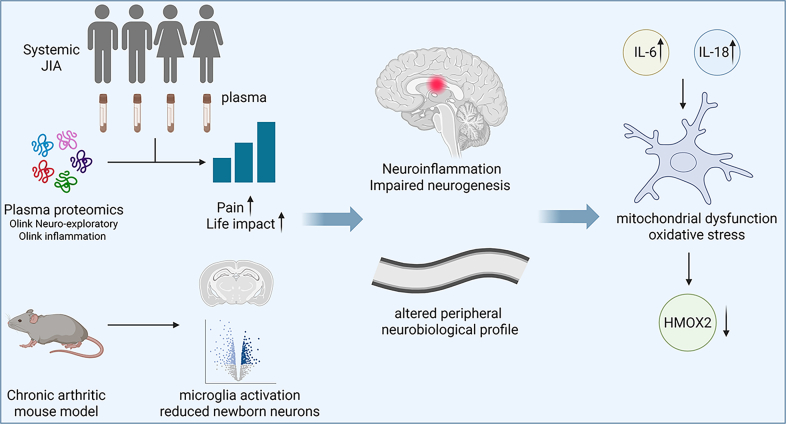
**Graphic abstractillustratingneurobiological alterations during chronic arthritis**.

To elucidate the direct effects of IL-6 and IL-18 on microglial oxidative stress, we treated SimA9 microglia cells with IL-6, IL-18, or a combination of both. IL-6 treatment alone did not induce significant ROS production, whereas IL-18 stimulation resulted in a moderate increase in ROS levels. Strikingly, co-treatment with IL-6 and IL-18 elicited a synergistic effect, leading to a substantial increase in ROS production (Fig. 7D and E). Corresponding ELISA assays for HMOX2 revealed no significant change in HMOX2 expression following IL-6 treatment, a moderate reduction with IL-18 treatment, and a more pronounced decrease upon combined IL-6/IL-18 stimulation (Fig. 7F). Taken together, these findings suggest that IL-6 and IL-18, particularly IL-18, can synergistically induce oxidative stress in microglial cells, leading to downregulation of HMOX2 expression. This IL-6/IL-18-driven oxidative response may represent a potential mechanism underlying the neurobiological alterations observed during chronic arthritis (Fig. 8).

## Discussion

Over the decades, clinicians have increasingly recognised challenges related to emotional well-being and cognitive functioning in children living with JIA.^7^^,^^37^ However, current assessments of these neuropsychological features in JIA patients are not common,^38^ and direct evidence of structural or functional alterations within the CNS in JIA patients remains limited.^39^ In this exploratory study, we demonstrate that patients with sJIA exhibit distinct neurobiological proteomic alterations in plasma. Reflecting the clinical observations, we identified neuroinflammation and impaired neurogenesis in the hippocampus of arthritic mice, demonstrating that CNS neuroinflammation is induced during chronic experimental arthritis. Lai et al. also reported the CNS changes in CIA rats.^40^ These findings provide molecular evidence supporting the recognised but mechanistically unexplained association between chronic arthritis and neuropsychiatric symptoms in individuals with JIA.

We identified an altered neurobiological protein profile in patients with sJIA, with 32 proteins downregulated and 1 protein (PAEP) upregulated. Notably, a recent study on Prader–Willi syndrome found a similar pattern, with 24 out of 29 differentially expressed proteins decreased, including several overlapping proteins like HMOX2, NAA10, FKBP5, TBCB, and PTPN1.^41^ These findings, along with a Mendelian randomisation analysis by Reppeto et al., which linked lower plasma levels of approximately 75% of 184 proteins from the Olink Neurology and Olink Neuro Exploratory panels to increased risk of adverse outcomes with neuropsychiatric and non-neurological comorbidities,^42^ further suggest that reductions in circulating neurologically related proteins reflect biologically meaningful changes in the nervous system. We also identified two distinct neurobiological protein profile clusters among patients with sJIA. Patients in Cluster 2 exhibited a more markedly altered neurobiological profile, along with higher pain levels and greater life impact, compared with Cluster 1. This further supports that alteration in the neurobiological profile reflects the patients neurofunctional status to some extent. Further, our paired analysis revealed that even when patients transitioned from the active to the inactive phase, neurobiological alterations persisted, suggesting possible lasting effects on the nervous system. This might explain why patients in Cluster 2 have a longer disease duration as compared to patients in Cluster 1. Consistently, a recent study reported sustained hippocampus neuroinflammation in JIA patients even during inactive disease.^13^

Our study also highlights the critical role of elevated peripheral IL-6 and IL-18 in hippocampal neuroinflammation during chronic arthritis. IL-6 and IL-18 have been reported to contribute to neuroinflammation by promoting BBB disruption and astrocyte and microglial activation, and IL-18 also exerts potent effects on the CNS by amplifying neurotoxic cascades.^43^, ^44^, ^45^ We observed positive correlations between serum IL-6 and IL-18 levels (the correlation with IL-6 did not reach statistical significance) and microglial activation in the hippocampus in arthritic mice, indicating that elevated levels of these cytokines could be contributing factors to the central neuroinflammatory processes detected. *In vitro* experiments further elucidated the distinct and synergistic effects of these cytokines. In sJIA, we found that both IL-6 and IL-18 levels were significantly elevated in patients with active disease. Notably, while IL-6 levels normalised following treatment, IL-18 levels remained persistently elevated in patients who had entered the inactive phase. Another study also reported elevated IL-18 levels in patients with inactive sJIA, even among those receiving IL-1 inhibitors.^46^ This residual elevation of IL-18 may partially explain the ongoing neurobiological protein profile changes observed in patients with inactive sJIA, despite the apparent clinical remission of peripheral symptoms. Current therapeutic strategies primarily focus on IL-1 and IL-6 blockade in sJIA, which effectively controls systemic inflammation and joint manifestations.^47^ However, the persistent elevation of IL-18 may represent a key driver of unresolved neurobiological alterations in these patients. Our study suggests that future therapeutic approaches should also consider targeting IL-18 to achieve a more comprehensive suppression of neuroinflammatory pathways during chronic arthritis.

HMOX2 was identified as a potential peripheral biomarker in our study, reflecting central neuroinflammation during chronic arthritis. HMOX2 is a haem oxygenase isoform that participates in haem degradation, redox regulation, and neuroprotection, and is enriched in the brain and testis.^31^^,^^48^ In this study, we consistently observed a significant reduction of peripheral HMOX2 levels in both patients with sJIA and in arthritic mice. The expression of HMOX2 in hippocampus was also reduced in arthritic mice. Notably, plasma HMOX2 expression was inversely correlated with clinical assessments of pain intensity and life impact scores in patients. In arthritic mice, lower serum HMOX2 levels correlated with increased microglial activation in the hippocampus, indicating a connection between peripheral HMOX2 expression and CNS neuroinflammatory status. Similarly, in the APP/PS1 transgenic mouse model of Alzheimer's disease, HMOX2 expression was also found to be decreased both in the hippocampus^49^ and in serum,^50^ further supporting a relevance of HMOX2 in neuroinflammatory contexts. Based on these observations, peripheral HMOX2 levels may serve as a promising indicator of CNS neuroinflammation in chronic arthritis. Meanwhile, we observed that the pro-inflammatory cytokines IL-6 and IL-18 were elevated in arthritic mice and serum IL-18 levels positively correlated with microglial activation in the hippocampus. However, considering that IL-6 and IL-18 are pleiotropic cytokines and their upregulation is a common feature of various cell types and tissues,^51^^,^^52^ their specificity for indicating CNS neuroinflammation is limited. In contrast, HMOX2 may confer a higher specificity for reflecting neuroinflammatory changes within the CNS. However, it should be noted that peripheral cells, such as endothelial cells, also express HMOX2.^53^ Currently, it is not clear whether decreased plasma HMOX2 levels are affected by CNS HMOX2 levels, or whether CNS HMOX2 can cross the BBB and contribute to peripheral HMOX2 levels. Therefore, it is difficult to definitively attribute altered peripheral HMOX2 expression solely to microglial activation. Further investigation into the regulation of HMOX2 at both peripheral and central levels, as well as their potential association, is required. Nevertheless, we propose that a reduction in peripheral HMOX2 levels may reflect both systemic and CNS oxidative stress and might be useful as a proxy for arthritis-associated neuroinflammation.

Several limitations of this study should be acknowledged. Firstly, the CIA mouse model, which is widely utilised to mimic Rheumatoid arthritis, was employed in this study. Although CIA is not an ideal model for sJIA and differs from sJIA in specific immunological mechanisms, both conditions share features such as elevated systemic inflammation, activation of immune cells, and joint involvement.^54^^,^^55^ Given the current lack of an appropriate experimental model that fully recapitulates sJIA pathology, we selected the CIA model to emphasise the chronicity of arthritis and its neuroinflammatory consequences, recognising this as a necessary compromise. Additionally, differences in the biological maturity between the mice used for CIA and the human sJIA study population should also be considered as a potential limitation. Secondly, psychometric data were not available for the patient cohort, limiting our ability to directly evaluate neuropsychiatric outcomes. Consequently, we utilised pain and life impact scores as surrogate indicators of neurological involvement. However, it is important to note that these surrogate indicators are also influenced by a range of factors, including social and psychological factors,^56^ and may not exclusively reflect neurobiological changes. Standardised psychiatric assessments and neuroimaging techniques such as functional MRI (fMRI), particularly targeting hippocampal activity are needed to enable a more objective and precise characterisation of neurobiological alterations in future studies. Thirdly, while our findings suggest a synergistic role of IL-6 and IL-18 in promoting neuroinflammation, these observations require further *in vivo* validation to confirm their mechanistic relevance and therapeutic potential.

In summary, this study identified alterations in neurobiological protein profiles in patients with sJIA. Even in an inactive disease phase, although inflammation and disease activity were reduced, persistent neurobiological changes remained, suggesting potential long-term implications for affected individuals. In chronic arthritic mice, we observed hippocampal neuroinflammation and increased oxidative stress. Notably, HMOX2 emerged as a potential peripheral biomarker of CNS neuroinflammation, with reduced levels significantly correlating with clinical measures of pain and life impact in patients, as well as with hippocampal microglial activation in mice. Furthermore, we found that IL-6 and particularly IL-18 play critical roles in neuroinflammation during arthritis, underscoring their relevance as a potential target for future therapeutic strategies.

## Contributors

Study conception and design: Xingzhao Wen, Heshuang Qu, Cecilia Aulin, and Helena Erlandsson Harris. Acquisition, analysis, and interpretation of the data: Xingzhao Wen, Malgorzata Benedyk-Machaczka, Daphne Chen, Cecilia Aulin, Erik Sundberg, Heshuang Qu, Maria Altman, and Helena Erlandsson Harris. Patient recruitment and sample collection: Erik Sundberg, Erik Melén, Cecilia Aulin, Maria Altman, and Helena Erlandsson Harris. Manuscript drafting and editing: Xingzhao Wen, Cecilia Aulin, and Helena Erlandsson Harris. Critical revision of the article for important intellectual content: Heshuang Qu, Malgorzata Benedyk-Machaczka, Daphne Chen, Erik Sundberg, Erik Melén, and Maria Altman. Xingzhao Wen, Cecilia Aulin, and Helena Erlandsson Harris have accessed and verified the underlying data. All authors read and approved the final version of the manuscript.

## Data sharing statement

The raw RNA-sequencing data from mouse hippocampal tissues have been deposited in the Sequence Read Archive (SRA) under accession number PRJNA1348353. Other original data and materials are available from the corresponding author upon reasonable request.

## Equitable partnership declaration

The authors of this paper have submitted an Equitable Partnership Declaration (attached in Supplementary Materials).

## Declaration of interests

The authors declare no conflicts of interest.

## Appendix A Supplementary data

Supplementary Tables and Figures

Western Blot

Equitable Partnership Declaration
