# Supplementary material for: Hippocampal neuroinflammation and altered peripheral neurobiological protein profile in experimental arthritis and systemic juvenile idiopathic arthritis
Source: eBioMedicine. 2026 Jun 16;129:106330. doi: 10.1016/j.ebiom.2026.106330 (PMC13292667; doi:10.1016/j.ebiom.2026.106330)
Supplement: Supplementary Tables and Figures [file mmc1.pdf]

Supplementary Materials

Content

Supplementary Figures 2
Figure S1. 2
Figure S2 3
Figure S3. 4
Figure S4. 5
Supplementary Tables 6
Table S1 6
Table S2. 6
Table S3 6
Table S4 8
Table S5 10
Table S6 11
Table S7 11
Table S8 12
Table S9 12
Table S10 14

Supplementary Figures

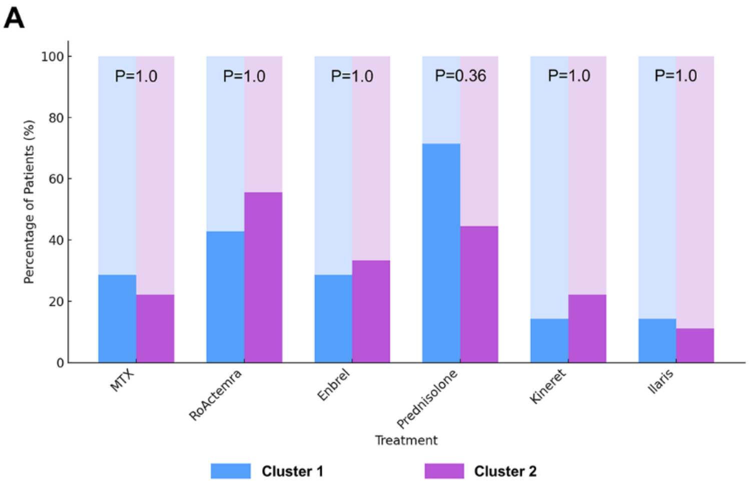

Figure S1. Analysis on the treatment in sJIA patients between two clusters. (A) Box plots showing the frequency of different treatments (Enbrel, Ilaris, Kineret, MTX, Prednisolone, and RoActemra) in two clusters of sJIA patients. Statistics: (A) Fisher's exact test to compare the differences in drug usage frequency between the two clusters.

A

Mouse	Total arthritis score	Arthritis duration(d)	Days with arthritis score >2
1	56	33	28
2	64	45	11
3	41	37	24
4	40	31	31
5	68	49	33
6	39	26	20

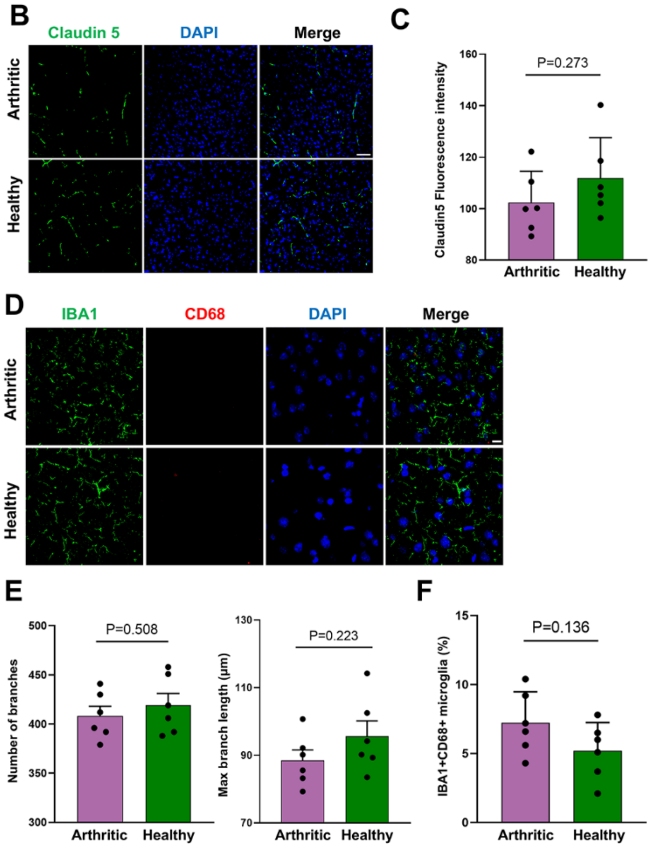

Figure S2. **Chronic arthritic mice do not exhibit significant cortical changes compared with healthy mice. (A)** Detailed arthritis assessment for each mouse (recorded from the booster injection, with 20 time points as referenced in Figure 2A). **(B)** Representative images of IF staining for Claudin-5 in the cortex of arthritic (n = 6) and healthy (n = 6) mice. Scale Bar: 100μm. **(C)** Box plots quantifying Claudin-5 fluorescence intensity by IF in arthritic and healthy mice. **(D)** Representative images of IF staining for microglial markers (IBA1 and CD68) in the cortex of arthritic and healthy mice. Scale Bar: 10μm. **(E)** Box plots quantifying microglial morphology (branch number and branch length) in arthritic and healthy mice. **(F)** Box plots showing the number of activated microglial cells ($IBA1^{+}$ $CD68^{+}$) by IF in arthritic and healthy mice. Statistics: (C, E, F) unpaired t-test.

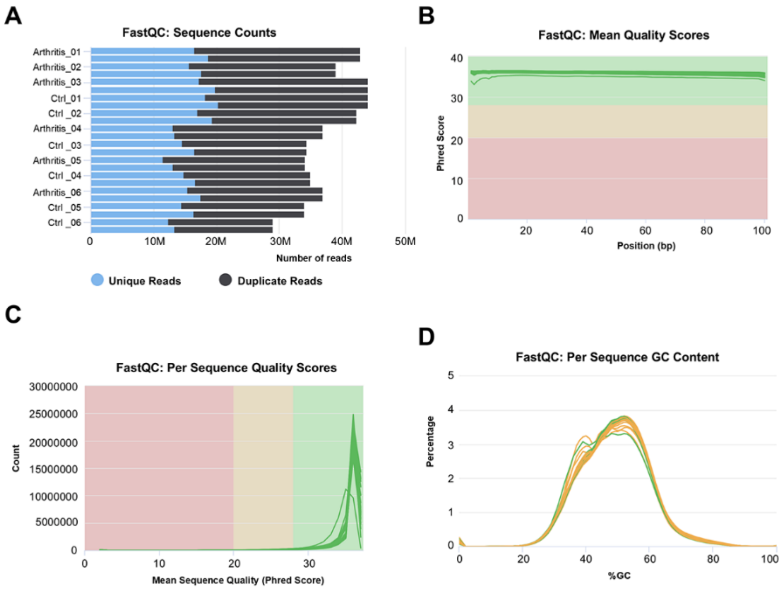

Figure S3. Overview of RNA sequence quality assessed using FastQC and MultiQC. (A) Raw sequence counts for each sample, distinguishing between duplicate and unique reads. **(B)** Per-base Phred quality scores, averaged across all bases in each read, indicating high-quality sequencing. **(C)** Per-sequence Phred quality scores, demonstrating that most reads have a quality >30, confirming their reliability. **(D)** Per-sequence GC content plot, showing a normal distribution of GC content across all sequencing reads.

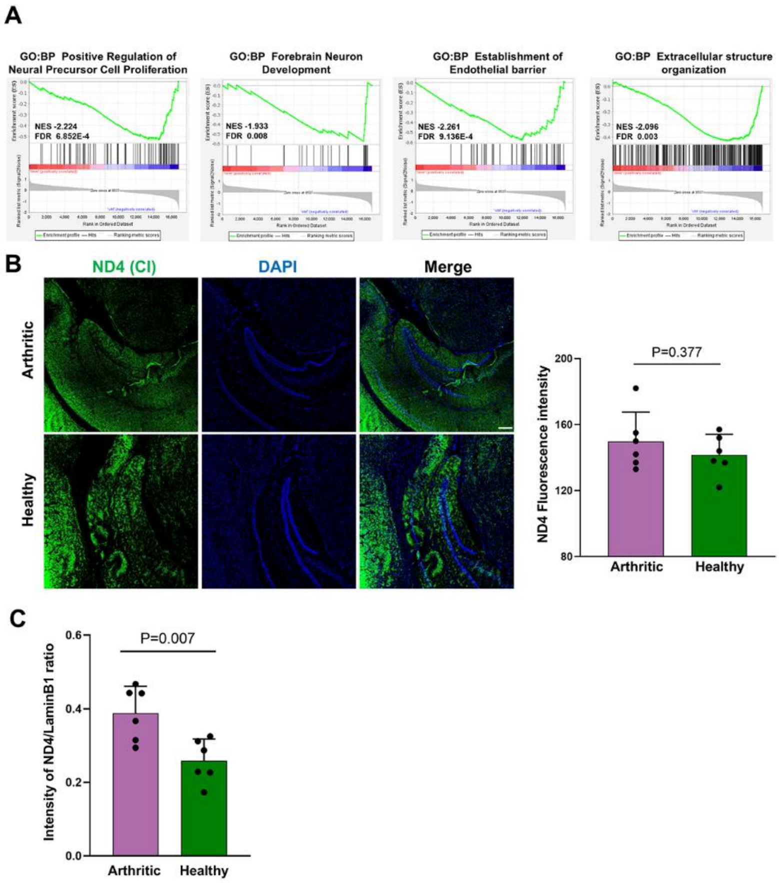

Figure S4. Downregulated pathways and mt-ND4 expression in the hippocampus of arthritic mice compared with Healthy mice. (A) GSEA plots showing inhibition of pathways related to neurogenesis and blood–brain barrier integrity in arthritic mice. **(B)** Representative images of IF staining for mt-ND4 in the hippocampus of arthritic (n = 6) and healthy (n = 6) mice, with corresponding box plot quantifying mt-ND4 fluorescence intensity. Scale Bar: 200μm. **(C)** Box plots quantifying mt-ND4 grayscale intensity normalized to Lamin B1 by western blotting (Figure 3E) in arthritic and healthy mice. Statistics: (B, C) unpaired t-test.

Supplementary Tables

Table S1. Demographics and disease characteristics of the study population

	sJIA patients	Healthy controls
Sample size(n)	16	16
Sex at birth (Female/Male)	8/8	8/8
Age at sampling (median/range)	9.0 (3.0-16.0)	8.0 (4.0-12.0)
**CRP** Measurement(n)	16	
median/range (mg/L)	35 (1-193)	
**RF** Measurement(n)	11	
positive	0 (0.00%)	
**ANA** Measurement(n)	14	
positive	2 (14.29%)	
**HLA-B27** Measurement(n)	9	
positive	1 (11.11%)	
cJADAS-71	8.00±2.06*	
VAS pain	4.39±2.45*	
Life impact	3.65±2.11*	

*: 2 patients lack the scores of these clinical parameters, resulting in 14 patients measured in total.

Abbreviation: C-reactive protein (CRP); Rheumatoid factor (RF); Antinuclear antibodies (ANA); Clinical Juvenile Arthritis Disease Activity Score of 71 points (cJADAS-71); patient visual analogue scale of pain (VAS pain,0-10); patient visual analogue scale of well-being (Life impact,0-10)

Table S2. Antibodies used in this study

Antibodies	Brand and catalog number	Work concentration
**Primary antibodies (IF)**		
Claudin5	Invitrogen, 34-1600	1:250
IBA1	Abcam, ab5076	1:300
CD68	Abcam, ab125212	1:250
DCX	Abcam, ab18723	1:300
Mt-ND4	Invitrogen, PA5-116791	1:300
NOX2	Invitrogen, PA5-79118	1:100
4-HNE	Abcam, ab46545	1:200
HMOX2	Sigma, SAB2501278	1:300
P2ry12	Cell Signaling Technology, 69766	1:300
**Primary antibodies (WB)**		
IBA1	Abcam, ab5076	1:800
DCX	Abcam, ab18723	1:800
Mt-ND4	Invitrogen, PA5-116791	1:1000
HMOX2	Sigma, SAB2501278	1:1000
Lamin B1	Cell Signaling Technology, 12586	1:1500
**Secondary antibodies (IF)**		
Alexa Fluor® 488 donkey anti-goat IgG (H+L)	Invitrogen, A-11055	1:1000
Alexa Fluor® 647 donkey anti-goat IgG (H+L)	Invitrogen, A-21447	1:1000
Alexa Fluor® 647 donkey anti-rabbit IgG (H+L)	Invitrogen, A-31573	1:1000
Alexa Fluor® 488 goat anti-rabbit IgG (H+L)	Invitrogen, A-11008	1:1000
**Secondary antibodies (WB)**		
HRP conjugate-goat anti-rabbit IgG (H + L)	Bio-Rad, 1706515	1:2000
HRP conjugate-rabbit anti-goat Immunoglobulins	Dako, P0449	1:2000

Table S3. Comparison of neurological protein expression between sJIA and HC in plasma

Protein	ΔNPX (sJIA-HC) / log2 Fold Change	p_value	FDR_BH
IMPA1	-2.27492	9.98E-08	7.27E-06
ABHD14B	-2.41557	2.02E-07	7.27E-06
PPP3R1	-2.09976	2.62E-07	7.27E-06
FHIT	-1.84467	3.20E-07	7.27E-06
COL4A3BP	-2.27924	4.40E-07	8.01E-06
DPEP2	-1.42284	5.41E-07	8.21E-06

CRADD	-2.66881	7.52E-07	9.77E-06
RPS6KB1	-2.40402	1.45E-06	1.65E-05
UBE2F	-0.93519	2.95E-06	2.73E-05
NAA10	-2.92807	3.00E-06	2.73E-05
ATP6V1F	-1.90691	6.05E-06	5.01E-05
HMOX2	-2.32116	7.21E-06	5.47E-05
AKT1S1	-1.37359	1.05E-05	6.65E-05
KIF1BP	-2.22766	1.08E-05	6.65E-05
FKBP7	-1.13078	1.10E-05	6.65E-05
WWP2	-1.03245	1.21E-05	6.65E-05
CETN2	-1.83528	1.24E-05	6.65E-05
TBCB	-2.86999	1.35E-05	6.83E-05
PRTFDC1	-2.54410	2.03E-05	9.74E-05
FGFR2	-0.39734	2.66E-05	0.000121
ILKAP	-2.05154	2.88E-05	0.000125
PSME1	-1.32593	7.77E-05	0.000322
CD63	-1.26003	0.000110	0.000435
GPNMB	-0.19784	0.000189	0.000715
SMOC1	0.82712	0.000225	0.000819
ECE1	-1.02307	0.000247	0.000859
PTPN1	-2.28161	0.000255	0.000859
FKBP5	-2.09816	0.000272	0.000883
EIF4B	-1.03023	0.000355	0.001113
ADAM15	-0.61646	0.000517	0.001568
DEFB4A	-2.08861	0.000651	0.001911
PMVK	-1.82161	0.000725	0.002060
SRP14	-2.11242	0.001040	0.002868
EREG	-1.47936	0.001120	0.002999
PFDN2	-0.57588	0.001393	0.003536
CD302	0.49330	0.001399	0.003536
MAD1L1	-1.97845	0.001641	0.004004
KIRREL2	-0.58651	0.001672	0.004004
LEPR	-0.43325	0.001826	0.004261
GSTP1	-0.91604	0.002904	0.006608
AOC1	-0.61463	0.003183	0.007064
ISLR2	-0.49595	0.003407	0.007234
IL32	-0.94637	0.003430	0.007234
ING1	-1.80630	0.003498	0.007234
KLB	-0.77197	0.003844	0.007774
CDH17	-0.85533	0.003955	0.007825
RNF31	-0.46339	0.004933	0.009552
BST2	-0.41492	0.005503	0.010433
DSG3	-0.45040	0.006241	0.011591
TPPP3	-0.40758	0.006665	0.012131
NPM1	-2.29931	0.007057	0.012592
SFRP1	-0.69263	0.007317	0.012804
CTF1	-0.43732	0.008207	0.013907
GGT5	-0.22193	0.008252	0.013907

ASGR1	0.24284	0.010252	0.016963
PAEP	1.27497	0.010464	0.017003
PTS	0.80530	0.012319	0.019667
AARSD1	-0.90518	0.016589	0.026027
DUSP3	-0.75734	0.022460	0.034641
VSTM1	0.51867	0.029126	0.044174
CDH15	-0.83234	0.030238	0.045108
NDRG1	-0.44972	0.036639	0.053777
IL15	0.34641	0.041896	0.060516
CD33	0.53352	0.058821	0.083637
TNFRSF13C	-0.31789	0.071110	0.099554
ADGRB3	-0.13974	0.075257	0.103763
IL3RA	-0.21028	0.084794	0.115168
CCL27	-0.24777	0.091647	0.122645
RBKS	-0.52251	0.110488	0.145716
CLSTN1	-0.23102	0.120208	0.156270
FCAR	0.37717	0.136439	0.174873
PHOSPHO1	-0.12624	0.153056	0.193445
PSG1	0.34535	0.168370	0.209886
CEACAM3	-0.11706	0.175144	0.215380
PLA2G10	-0.33581	0.226109	0.274345
TDGF1	-0.67336	0.274401	0.328559
DPEP1	0.20243	0.302282	0.357243
IFI30	-0.40804	0.315414	0.367983
IFNL1	-0.14939	0.391039	0.450437
NEFL	0.11106	0.407968	0.464063
IKZF2	0.31818	0.452960	0.508881
FUT8	-0.12450	0.518434	0.575336
HSP90B1	0.10192	0.533327	0.584732
CRIP2	-0.09166	0.585899	0.634723
LTBP3	0.04523	0.649544	0.695394
KIR2DL3	0.08150	0.674287	0.713490
ANXA10	-0.11302	0.693869	0.725771
SNCG	-0.04925	0.727562	0.752365
EPHA10	0.08658	0.873543	0.893174
CARHSP1	-0.00489	0.982990	0.985944
NXPH1	0.00357	0.985944	0.985944

Table S4. Comparison of neurological protein expression between cluster 1 and cluster 2 sJIA patients

Protein	ΔNPX (C2-C1) / log2 Fold Change	p_value	FDR_BH
FKBP5	-3.15969	5.47E-05	0.003806
CDH15	1.38183	8.36E-05	0.003806
KIF1BP	-2.15189	0.000357	0.00778
PRTFDC1	-2.70749	0.00037	0.00778
NAA10	-2.46039	0.000427	0.00778

PTPN1	-2.54681	0.000696	0.008846
TBCB	-2.87358	0.000833	0.008846
HMOX2	-2.02248	0.000849	0.008846
ILKAP	-1.42180	0.000875	0.008846
CETN2	-1.47148	0.001199	0.010913
PMVK	-2.42226	0.001459	0.011357
COL4A3BP	-1.63856	0.001498	0.011357
EIF4B	-1.34765	0.001847	0.012928
KIRREL2	0.79299	0.002846	0.018496
CD63	-1.45705	0.004052	0.023703
ASGR1	-0.31189	0.004384	0.023703
FGFR2	0.32265	0.004428	0.023703
ECE1	-0.90944	0.006725	0.034
DSG3	0.68236	0.007684	0.036716
WWP2	-1.25308	0.008069	0.036716
AKT1S1	-1.48778	0.009422	0.038497
RPS6KB1	-1.33026	0.009559	0.038497
PFDN2	-0.50155	0.00973	0.038497
ADGRB3	0.34599	0.020027	0.075937
MAD1L1	-0.96021	0.021399	0.077891
CRADD	-1.36491	0.025032	0.087612
TNFRSF13C	0.46731	0.026643	0.089798
SFRP1	0.44015	0.028641	0.093084
FCAR	-0.89771	0.034182	0.107262
SMOC1	-0.74864	0.036257	0.109979
CCL27	-0.47898	0.040185	0.117964
VSTM1	-0.79491	0.041677	0.11852
FHIT	-0.75313	0.047372	0.129113
RNF31	-0.40838	0.04824	0.129113
IL15	-0.37235	0.052748	0.137144
PPP3R1	-0.84201	0.056341	0.142417
IFNL1	0.46419	0.066517	0.163597
PSME1	-0.76100	0.069897	0.167385
FKBP7	-0.42956	0.075118	0.175275
DPEP2	0.40856	0.078134	0.177756
SRP14	-1.00918	0.096758	0.214756
EREG	-0.53400	0.119656	0.259254
IMPA1	-0.71611	0.133213	0.281917
GSTP1	-0.58532	0.138948	0.28737
IL32	0.57752	0.156257	0.315986
ATP6V1F	-0.49655	0.16342	0.316749
CD33	-0.50494	0.163596	0.316749
NPM1	-0.71688	0.176532	0.334675
ANXA10	-0.57284	0.192596	0.354331
AOC1	-0.43905	0.194687	0.354331
ING1	-0.35542	0.208023	0.367615
CDH17	0.546617	0.212994	0.367615
DEFB4A	-1.03223	0.214106	0.367615

CTF1	-0.22727	0.240576	0.399519
PHOSPHO1	0.18474	0.245695	0.399519
IKZF2	0.73201	0.245858	0.399519
EPHA10	-0.95448	0.265695	0.42418
IFI30	-0.36306	0.286625	0.442185
PTS	-0.70128	0.286692	0.442185
NXPH1	-0.50333	0.291838	0.442621
NDRG1	-0.27826	0.341255	0.50291
CD302	-0.13903	0.342642	0.50291
IL3RA	0.13916	0.35064	0.50648
BST2	0.17480	0.366323	0.520866
PAEP	0.79158	0.380773	0.533082
ABHD14B	-0.32838	0.396839	0.547156
CARHSP1	0.29779	0.450408	0.611748
GPNMB	-0.04845	0.534031	0.714659
AARSD1	-0.25229	0.59379	0.783114
DUSP3	-0.08316	0.61374	0.79661
GGT5	-0.06265	0.632626	0.79661
LTBP3	0.09932	0.634812	0.79661
ADAM15	0.10478	0.639039	0.79661
HSP90B1	0.10767	0.650809	0.800319
FUT8	-0.11978	0.667112	0.809429
PLA2G10	-0.14968	0.71268	0.853341
UBE2F	-0.03576	0.735376	0.864507
TDGF1	-0.25750	0.750495	0.864507
RBKS	-0.15239	0.750506	0.864507
CEACAM3	0.01692	0.762485	0.867327
NEFL	-0.05875	0.794499	0.890698
LEPR	0.04657	0.809352	0.890698
SNCG	-0.05494	0.812395	0.890698
PSG1	0.08620	0.832859	0.902264
ISLR2	0.05523	0.844126	0.903712
CRIP2	0.03306	0.905755	0.953429
DPEP1	0.03222	0.91152	0.953429
CLSTN1	-0.01371	0.944907	0.977119
KIR2DL3	-0.01097	0.962133	0.983754
TPPP3	0.00768	0.976985	0.986244
KLB	0.00936	0.986244	0.986244

Table S5. Spearman correlation analysis results between proteins selected and Vas pain score in sJIA patients (ranked by P values)

Protein	Spearman_r	p_value	FDR_BH
HMOX2	-0.679121	0.007562	0.038862
FKBP5	-0.670330	0.008704	0.038862
KIF1BP	-0.670330	0.008704	0.038862
PRTFDC1	-0.652747	0.011385	0.038862

TBCB	-0.648352	0.012144	0.038862
CETN2	-0.600037	0.023298	0.057709
PTPN1	-0.586813	0.027384	0.057709
NAA10	-0.582418	0.028855	0.057709
COL4A3BP	-0.560440	0.037104	0.065964
CD63	-0.538462	0.046976	0.075162
PMVK	-0.525275	0.053748	0.076520
EIF4B	-0.512088	0.061198	0.076520
RPS6KB1	-0.510451	0.062172	0.076520
ILKAP	-0.483448	0.079885	0.091297
WWP2	-0.463736	0.094876	0.098468
AKT1S1	-0.459341	0.098468	0.098468

Table S6. Spearman correlation analysis results between proteins selected and life impact score in sJIA patients (ranked by P values)

Protein	Spearman_r	p_value	FDR_BH
HMOX2	-0.721673	0.003570	0.028504
KIF1BP	-0.706271	0.004753	0.028504
FKBP5	-0.699670	0.005345	0.028504
CETN2	-0.632957	0.015115	0.050891
TBCB	-0.629263	0.015903	0.050891
CD63	-0.607261	0.021269	0.052839
PRTFDC1	-0.600660	0.023117	0.052839
PTPN1	-0.580858	0.029390	0.053614
NAA10	-0.578658	0.030158	0.053614
COL4A3BP	-0.541254	0.045626	0.073001
ILKAP	-0.530390	0.051042	0.074243
WWP2	-0.514852	0.059579	0.079438
EIF4B	-0.497250	0.070438	0.086693
RPS6KB1	-0.469163	0.090569	0.100763
PMVK	-0.464247	0.094465	0.100763
AKT1S1	-0.389439	0.168708	0.168708

Table S7. Demographics and disease characteristics of two sJIA clusters

	Cluster 1	Cluster 2	P value
Sample size	7	9	-
Sex at birth (Female/Male)	2/5	6/3	0.315
Age at onset (median/range, year)	5.2 (2.7-15.0)	7.4 (0.7-13.8)	1.0
Age at sampling (median/range, year)	5.6 (3.4-16.9)	9.7 (3.1-15.2)	0.142
Disease activity score	7.20±2.37	8.60±1.71	0.222
Disease duration (median/range, year)	0.4 (0.1-2.3)	4.3(0.5-9.1)	0.003
**CRP** Measurement(n)	7	9	-
median/range (mg/L)	21 (1-123)	36 (1-193)	0.536
**RF** Measurement(n)	5	6	-
positive	0 (0.00%)	0 (0.00%)	-
**ANA** Measurement(n)	6	8	-
positive	2 (33.3%)	1 (12.5%)	1.0
**HLA-B27** Measurement(n)	4	5	-
positive	0 (0.00%)	1 (20.0%)	1.0
Treatments (On medication)			
Methotrexate	2 (28.57%)	2 (22.22%)	1.0
RoActemra (Tocilizumab)	3 (42.86%)	5 (55.56%)	1.0
Enbrel (Etanercept)	2 (28.57%)	3 (33.33%)	1.0
Kineret (Anakinra)	1 (14.29%)	2 (22.22%)	1.0

Ilaris (Canakinumab)	1 (14.29%)	1 (11.11%)	1.0
Prednisolone	5 (71.43%)	4 (44.44%)	0.358

Table S8. Top 30 pathways upregulated in arthritic mice (ranked by NES)

No.	GO:BP Pathways	ES	NES	FDR q value
1	AEROBIC_ELECTRON_TRANSPORT_CHAIN	0.80	3.78	0.000
2	TRANSLATION_AT_SYNAPSE	0.90	3.55	0.000
3	CYTOPLASMIC_TRANSLATION	0.72	3.55	0.000
4	MITOCHONDRIAL_TRANSLATION	0.64	3.49	0.000
5	MITOCHONDRIAL_GENE_EXPRESSION	0.62	3.32	0.000
6	RIBOSOMAL_SMALL_SUBUNIT_BIOGENESIS	0.63	3.22	0.000
7	PROTON_MOTIVE_FORCE_DRIVEN_ATP_SYNTHESIS	0.80	3.20	0.000
8	OXIDATIVE_PHOSPHORYLATION	0.67	3.19	0.000
9	AEROBIC_RESPIRATION	0.61	3.18	0.000
10	CELLULAR_RESPIRATION	0.54	3.10	0.000
11	NADH_DEHYDROGENASE_COMPLEX_ASSEMBLY	0.75	3.10	0.000
12	MITOCHONDRIAL_RESPIRATORY_CHAIN_COMPLEX_ASSEMBLY	0.66	3.06	0.000
13	ELECTRON_TRANSPORT_CHAIN	0.61	2.98	0.000
14	NUCLEOSIDE_TRIPHOSPHATE_BIOSYNTHETIC_PROCESS	0.62	2.97	0.000
15	RIBOSOME_BIOGENESIS	0.54	2.93	0.000
16	RIBONUCLEOPROTEIN_COMPLEX_BIOGENESIS	0.50	2.91	0.000
17	MITOCHONDRIAL_ELECTRON_TRANSPORT_NADH_TO_UBIQUINONE	0.83	2.90	0.000
18	RIBOSOMAL_LARGE_SUBUNIT_BIOGENESIS	0.64	2.75	0.000
19	PROTEIN_TARGETING_TO_MITOCHONDRION	0.57	2.66	0.000
20	MITOCHONDRIAL_RNA_METABOLIC_PROCESS	0.61	2.66	0.000
21	RIBOSOMAL_SMALL_SUBUNIT_ASSEMBLY	0.78	2.60	0.000
22	ENERGY_DERIVATION_BY_OXIDATION_OF_ORGANIC_COMPOUNDS	0.43	2.59	0.000
23	RIBOSE_PHOSPHATE_BIOSYNTHETIC_PROCESS	0.47	2.57	0.000
24	NUCLEOSIDE_TRIPHOSPHATE_METABOLIC_PROCESS	0.50	2.56	0.000
25	RIBOSOME_ASSEMBLY	0.62	2.53	0.000
26	PROTEIN_RNA_COMPLEX_ORGANIZATION	0.47	2.49	0.000
27	NUCLEOSIDE_PHOSPHATE_BIOSYNTHETIC_PROCESS	0.42	2.42	0.000
28	PROTEIN_IMPORT_INTO_MITOCHONDRIAL_MATRIX	0.76	2.41	0.000
29	MITOCHONDRION_ORGANIZATION	0.40	2.41	0.000
30	MITOCHONDRIAL_TRANSPORT	0.47	2.40	0.000

Table S9. Comparison of inflammation protein expression between sJIA and healthy individuals (ranked by P values)

Protein	ΔNPX (sJIA-HC) / log2 Fold Change	p_value	FDR_BH
IL6	3.61031	2.09E-07	1.44E-05
OSM	1.93935	2.24E-05	0.000578
TWEAK	-0.60175	2.95E-05	0.000578
CD5	-0.85901	3.35E-05	0.000578
TNFRSF9	-0.92278	4.28E-05	0.000591
CD6	-1.07644	0.000102	0.001168

MMP-1	1.91001	0.000579	0.00525
SCF	-1.28315	0.000609	0.00525
EN-RAGE	2.26512	0.000806	0.006182
CSF-1	0.24095	0.001279	0.008247
IL-18R1	0.58864	0.001315	0.008247
MMP-10	-0.65784	0.001813	0.010423
Flt3L	-0.57402	0.00235	0.012472
IL18	1.97081	0.003022	0.014893
DNER	-0.30138	0.003715	0.017088
TGF-alpha	0.31188	0.00456	0.019663
CCL23	0.62904	0.005194	0.021081
CXCL6	-1.25649	0.006198	0.02376
AXIN1	-1.08277	0.007014	0.025473
ST1A1	-1.11570	0.008214	0.027137
HGF	0.53089	0.008259	0.027137
CDCP1	0.48592	0.015035	0.047156
CASP-8	-1.02685	0.016517	0.049551
MCP-1	0.47187	0.023728	0.068217
IL-12B	-0.90411	0.027578	0.076114
FGF-21	1.20407	0.031377	0.083269
IL7	-0.71275	0.047716	0.12194
CD244	-0.34671	0.057056	0.140601
CCL19	-0.53534	0.059941	0.142618
NT-3	-0.35086	0.066659	0.153315
CCL25	-0.33152	0.078907	0.175632
IL-17A	0.54231	0.10701	0.23074
IL10	0.52394	0.118612	0.248007
MCP-4	-0.59654	0.134077	0.272098
uPA	-0.36863	0.142213	0.280362
CD8A	0.36739	0.159586	0.305872
TNFB	-0.51839	0.167709	0.312755
TRANCE	-0.92792	0.190314	0.34557
MCP-2	-0.44523	0.231279	0.409186
CX3CL1	-0.23189	0.240826	0.415424
CXCL5	-0.56436	0.260278	0.438029
CD40	-0.22450	0.27533	0.452328
STAMBP	-0.49348	0.287573	0.454841
IL-10RA	0.35208	0.294776	0.454841
VEGFA	0.30271	0.296635	0.454841
ADA	-0.31895	0.305728	0.458593
LAP TGF-beta-1	-0.22785	0.404649	0.594059
4E-BP1	0.38957	0.418868	0.602123
CCL3	0.22628	0.447075	0.629555
PD-L1	0.12878	0.456909	0.630535
LIF-R	0.06116	0.514484	0.6923
CXCL9	0.19376	0.521733	0.6923
FGF-19	-0.20881	0.54104	0.704373
SIRT2	-0.34184	0.552965	0.706566

CCL20	-0.13996	0.633569	0.794841
IL-10RB	-0.04515	0.701675	0.864563
TNFSF14	0.10646	0.751056	0.887644
TRAIL	-0.04478	0.751955	0.887644
FGF-23	0.06200	0.759	0.887644
CCL4	0.07142	0.805708	0.926564
CCL11	-0.03940	0.834	0.927593
CXCL1	-0.08076	0.835922	0.927593
CCL28	-0.02496	0.858503	0.927593
IL8	-0.04732	0.860376	0.927593
CST5	0.01868	0.912796	0.939951
IL-15RA	0.00807	0.922829	0.939951
CXCL10	0.03096	0.937208	0.939951
CXCL11	0.04426	0.937365	0.939951
OPG	0.01037	0.939951	0.939951

Table S10. Comparison of inflammation protein expression between Cluster 1 and Cluster 2 sJIA patients (ranked by P values)

Protein	ΔNPX (C2-C1) / log2 Fold Change	p_value	FDR_BH
AXIN1	-2.47100	0.000026	0.001794
SCF	-1.78449	0.000071	0.002450
CD40	0.83039	0.000276	0.006348
IL-18R1	0.69142	0.000595	0.010264
TRAIL	-0.55381	0.001059	0.014614
PD-L1	0.66938	0.002421	0.027842
IL18	2.07280	0.002980	0.029374
VEGFA	0.91719	0.003897	0.033612
CCL20	0.99925	0.004818	0.036938
uPA	-0.49929	0.005425	0.037433
HGF	0.73642	0.006095	0.038221
IL6	2.39492	0.009356	0.053797
EN-RAGE	2.18710	0.012916	0.068552
IL7	0.82460	0.015036	0.074117
CD6	-0.49490	0.016364	0.075274
OSM	1.13747	0.018024	0.077730
IL10	0.69988	0.019209	0.077990
CXCL5	1.20233	0.025734	0.098641
IL8	0.55269	0.029811	0.108236
SIRT2	1.36926	0.031697	0.109355
LAP TGF-beta-1	0.59808	0.034177	0.112296
MMP-1	1.18167	0.038002	0.119187
CXCL1	1.06217	0.042004	0.126012
Flt3L	-0.43693	0.046144	0.132664
CSF-1	0.16986	0.053551	0.147801
LIF-R	0.20539	0.065178	0.172978
CXCL11	1.13615	0.075893	0.193949
STAMBP	0.80765	0.084503	0.208240
TWEAK	-0.24288	0.092132	0.218855

CASP-8	0.58069	0.101131	0.232601
FGF-19	-0.58089	0.114143	0.254060
TNFB	-0.85174	0.127473	0.274862
TNFRSF9	-0.39569	0.135019	0.282312
CCL23	0.45112	0.144540	0.293337
ST1A1	0.61275	0.180125	0.355108
TNFSF14	0.50142	0.196993	0.377570
IL-12B	-0.39813	0.210854	0.393214
CX3CL1	-0.26473	0.235151	0.426986
IL-17A	-0.48136	0.259389	0.458919
CCL11	-0.23045	0.268691	0.463492
CXCL6	0.43072	0.279182	0.469837
MCP-1	-0.28882	0.291294	0.478554
MMP-10	0.17466	0.313130	0.501867
CCL3	0.33310	0.333277	0.522637
TRANCE	0.31855	0.366624	0.562158
CD5	-0.14091	0.382973	0.574460
OPG	0.13305	0.408998	0.599869
4E-BP1	-0.47340	0.420630	0.604656
TGF-alpha	0.09464	0.442046	0.622473
MCP-2	0.35545	0.454862	0.627710
CCL25	-0.12705	0.509170	0.688877
CCL28	0.06039	0.542778	0.720225
CD8A	-0.15728	0.555057	0.722943
MCP-4	0.22862	0.565789	0.722943
IL-10RA	0.20594	0.586517	0.735814
CXCL10	-0.26679	0.600923	0.740422
CXCL9	-0.14757	0.663005	0.802585
CCL19	-0.13994	0.674985	0.802585
DNER	0.04528	0.693019	0.808349
FGF-23	-0.09791	0.704771	0.810487
CD244	0.03204	0.754829	0.853284
CST5	-0.03583	0.771785	0.858597
FGF-21	-0.16380	0.796575	0.872438
IL-10RB	-0.02379	0.837826	0.903273
CDCP1	0.02557	0.900920	0.950970
NT-3	0.01151	0.931549	0.973893
ADA	-0.01182	0.955934	0.979528
CCL4	0.00646	0.974516	0.988715
IL-15RA	-0.00150	0.991690	0.991690
