# Supplementary figures and images for: Hippocampal neuroinflammation and altered peripheral neurobiological protein profile in experimental arthritis and systemic juvenile idiopathic arthritis
Source: eBioMedicine. 2026 Jun 16;129:106330. doi: 10.1016/j.ebiom.2026.106330 (PMC13292667; doi:10.1016/j.ebiom.2026.106330)

## Original western blot bands

Figure 3K

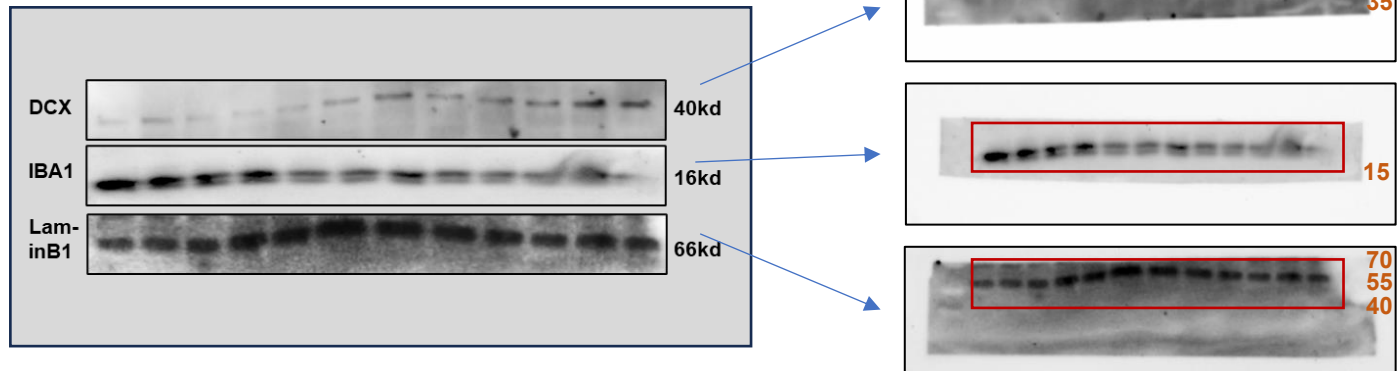

Figure 4F

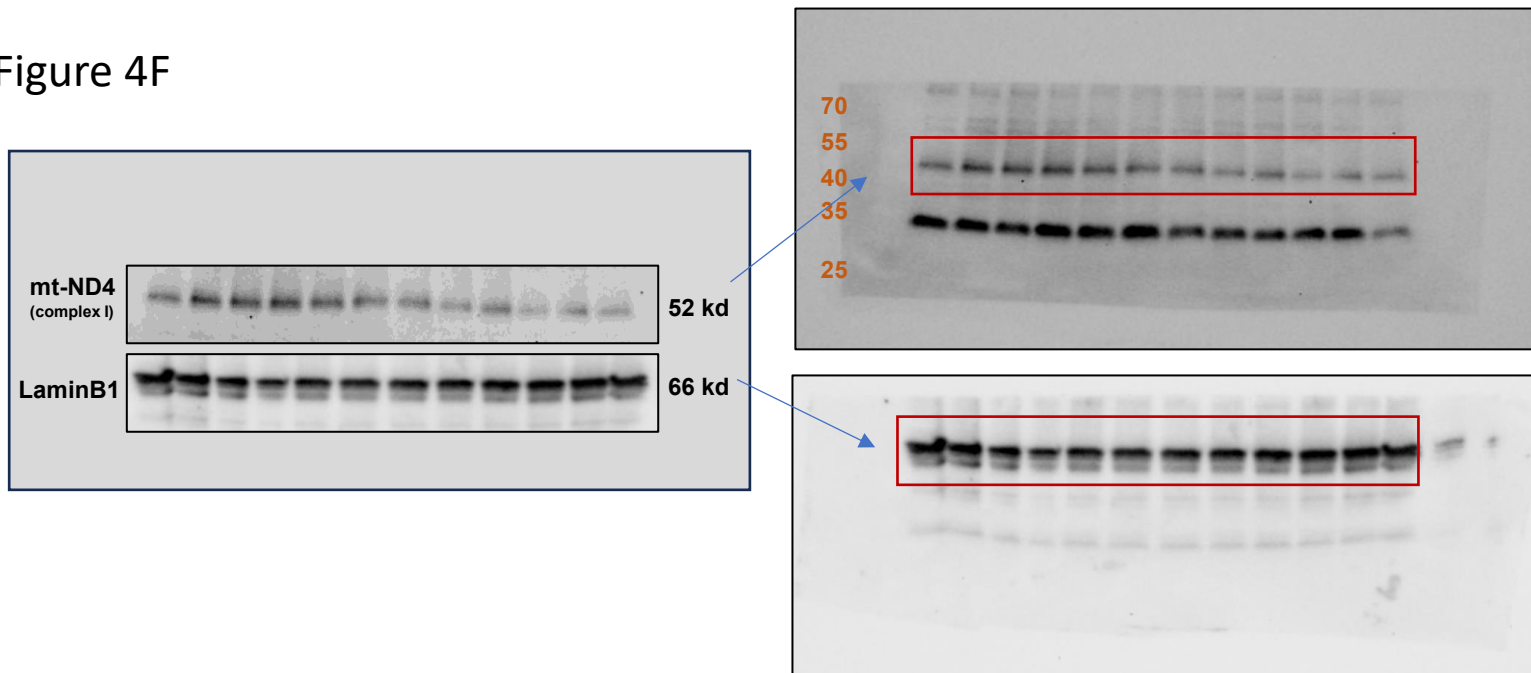

Figure 5D

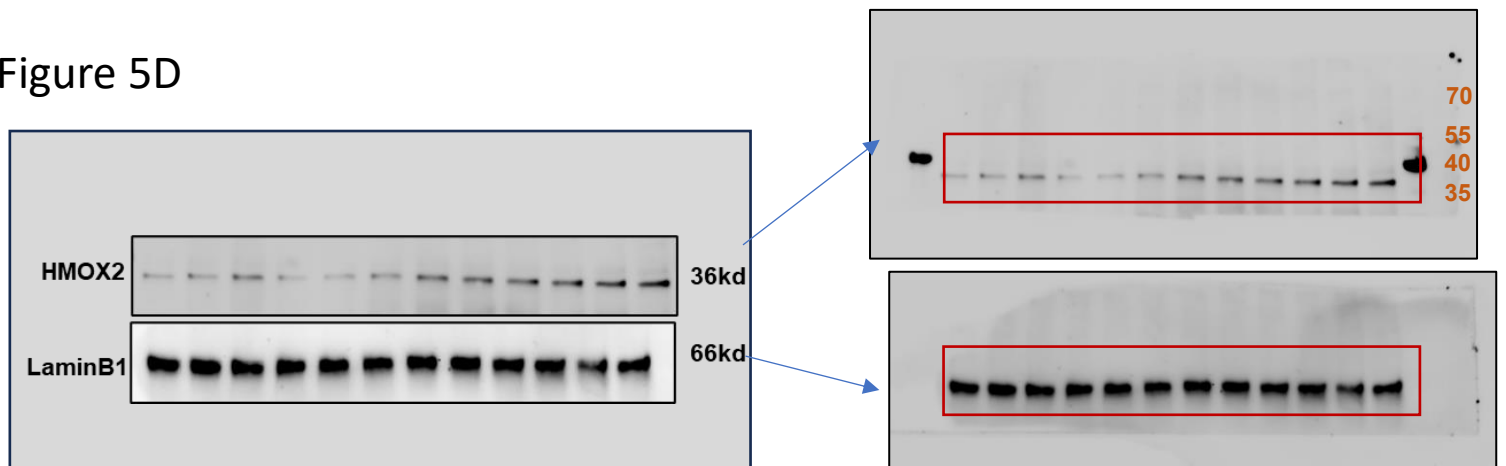

Figure 5J

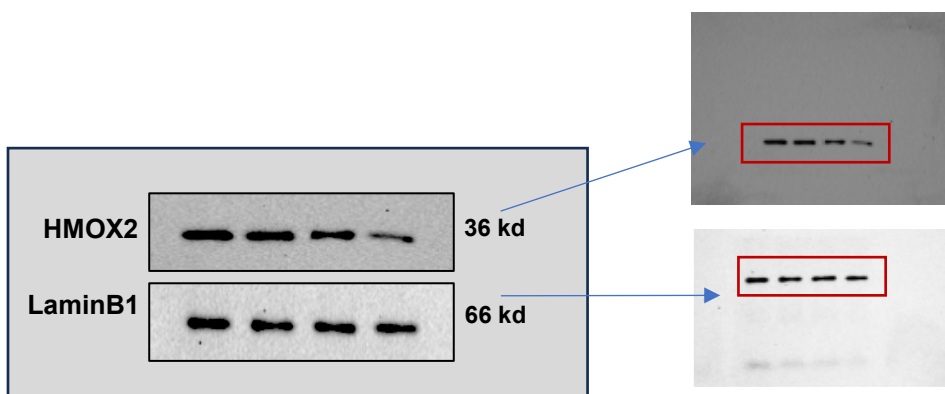

Supplement: Western Blot [file mmc2.pdf]
