# Supplementary material for: Hippocampal neuroinflammation and altered peripheral neurobiological protein profile in experimental arthritis and systemic juvenile idiopathic arthritis
Source: eBioMedicine. 2026 Jun 16;129:106330. doi: 10.1016/j.ebiom.2026.106330 (PMC13292667; doi:10.1016/j.ebiom.2026.106330)
Supplement: Equitable Partnership Declaration [file mmc3.docx]

**Equitable Partnership Declaration**

If any questions do not apply to your study, please indicate “N/A” for “not applicable.
For more information on how to complete this form see the Information for Authors document.

*Researcher considerations*

1. Please detail the involvement that researchers who are based in the country or countries of study had during a) study design; b) clinical study processes, such as processing blood samples, prescribing medication, or patient recruitment; c) data interpretation; and d) manuscript preparation, commenting on all aspects. If they were not involved in any of these aspects, please explain why.

*This should include a thorough description of their leadership roles in the study. Are local researchers named in the author list or the acknowledgements, or are they not mentioned at all (and, if not, why)? Please also describe the involvement of early career researchers based in the location of the study. Some of this information might be repeated from the Contributors section in the manuscript. Note: we adhere to* [*ICMJE authorship criteria*](https://www.icmje.org/recommendations/browse/roles-and-responsibilities/defining-the-role-of-authors-and-contributors.html) *for naming authors on a paper.*

| **a) Study design:**  Xingzhao Wen, Heshuang Qu, Cecilia Aulin, and Helena Erlandsson Harris were involved in the study conception, discussed the study design, and decided how to proceed with the study. |
| --- |
| **b) Clinical study processes:**  Erik Sundberg and Maria Altman collected plasma samples from patients with systemic JIA. Erik Melén provided plasma samples collected from healthy children. Xingzhao Wen, Cecilia Aulin, and Helena Erlandsson Harris processed the plasma samples and sent them for Olink proteomics analysis, after which Claudia Fredolini performed the Olink proteomics analysis. Malgorzata Benedyk-Machaczka conducted the arthritis model, including treatment and health screening. Xingzhao Wen, Heshuang Qu, and Daphne Chen investigated the changes in the arthritis mice. RNA-seq analysis was performed at the Genomics Core Facility (GCF) at the University of Bergen. |
| **c) Data interpretation:**  Xingzhao Wen and Heshuang Qu analysed the Olink proteomics data and the correlations between protein expression and clinical parameters. Xingzhao Wen, Heshuang Qu, and Daphne Chen investigated the brain molecular changes in arthritis mice using immunofluorescence staining and Western blot. Xingzhao Wen and Daphne Chen analysed the RNA-seq data and interpreted the results. |
| **d) Manuscript preparation:**  Xingzhao Wen, Cecilia Aulin, and Helena Erlandsson Harris prepared the preliminary version of the manuscript. All coauthors read it thoroughly and provided their suggestions. Xingzhao Wen revised the manuscript accordingly. All authors reviewed and approved the manuscript. |

1. How was funding used to remunerate and enhance the skills of researchers in the countries of study? And how was funding used to improve research infrastructure at the study sites?

*Potentially effective investments into long-term skills and opportunities within local institutions could include training or mentorship in analytical techniques and manuscript writing, opportunities to lead all or specific aspects of the study, financial remuneration rather than requiring volunteers, and other professional development and educational opportunities.*

*Improvements to research infrastructure could include funding extended trial designs (eg, platform trials), establishment of long-term contracts for research staff, building research facilities, and setting up local control of funding allocation.*

| **Skills:**  Funding supported the conduct of the study, including sample processing, proteomic analyses, animal experiments, and RNA-seq analysis. Researchers involved in the project contributed to data generation, analysis, interpretation, and manuscript preparation, which provided opportunities to further develop skills in proteomics data analysis, experimental techniques, and scientific writing. |
| --- |
| **Research infrastructure:**  The study was conducted within existing research infrastructure and core facilities, including established laboratory platforms and the Genomics Core Facility at the University of Bergen. No dedicated funding was allocated specifically to establish new research infrastructure at the study sites. |

1. How did you safeguard the researchers who implemented the study?

*Please describe how you guaranteed safe working conditions for study staff, including provision of appropriate personal protective equipment, protection from violence, and prevention of overworking.*

| To safeguard the researchers who implemented the study, several measures were put in place to ensure their safety and well-being. All researchers were provided with appropriate personal protective equipment, including lab coats, gloves, and safety goggles, to minimize exposure to hazardous materials. Comprehensive safety training was conducted to ensure that team members were fully aware of safety protocols, proper handling of samples and equipment, and emergency procedures. The work environment was regularly assessed for safety compliance, with adequate emergency response plans in place, including access to first-aid kits and fire extinguishers. To prevent overworking, work hours were closely monitored, and regular breaks were encouraged. Flexible scheduling was provided when necessary, ensuring researchers maintained a healthy work-life balance and were not overburdened by long hours. Furthermore, efforts were made to create a secure environment, with steps taken to protect staff from any potential harm or violence. Researchers were encouraged to report any concerns regarding their safety, both physical and emotional. These measures helped create a safe and supportive environment for all personnel involved in the study. |
| --- |

*Benefits to the communities and regions of study*

1. How does the study address the research and policy priorities of its location?

*How were the local priorities determined and then used to inform the research question? Who decided which priorities to take forward? Which elements of the study address those priorities?*

| The study was designed to address local research and policy priorities, which were identified through consultations with local clinicians and by recognizing gaps in current research. Clinical professionals highlighted the need for a better understanding of the neurological impacts of systemic juvenile idiopathic arthritis (sJIA), particularly in terms of how the disease affects brain function and development. Our research team identified this gap and developed the study to focus specifically on these neurological aspects of sJIA. The research question was shaped by these insights, aiming to explore the neurobiological changes in patients with sJIA. The decision on which priorities to pursue was made collaboratively by the research team in consultation with local clinicians, ensuring that the study addressed pressing clinical concerns. The study directly addresses these priorities by investigating the neurological effects of sJIA through various analyses, including the assessment of brain molecular changes in affected mice, with the goal of improving the understanding of how the disease impacts the brain and informing future clinical approaches to treatment. |
| --- |

1. How will research products be shared in the community of study?

*For instance, will you be providing written or oral layperson summaries for non-academic information sharing? Will study data be made available to institutions in the region(s) of study?* The Lancet Group *encourages authors to translate the summary (abstract) into relevant languages after paper editing; do you intend to translate your summary?*

| To ensure the research products are effectively shared within the community of study, we have developed a comprehensive dissemination plan. We will provide both written and oral layperson summaries to communicate the findings in an accessible way for the local community. These summaries will be tailored to ensure clarity and will be shared through community meetings, healthcare facilities, and local organizations to ensure broad understanding of the research outcomes. Additionally, we will collaborate with local institutions to make the study data available to them, fostering transparency and encouraging further research within the region. As part of our commitment to making the research accessible, we also plan to translate the summary (abstract) of the study into the relevant local languages after the paper editing process, in alignment with the suggestions of The Lancet Group. This will ensure that non-academic stakeholders can engage with the research findings, helping to bridge the gap between academia and the local community. |
| --- |

1. How were individuals, communities, and environments protected from harm?
   1. *How did you ensure that sensitive patient data were handled safely and respectfully? Was there any potential for stigma or discrimination against participants arising from any of the procedures or outcomes of the study?*

| We ensured that sensitive patient data were handled safely and respectfully by anonymizing and de-identifying all data before analysis, following ethical guidelines and data protection regulations. Access to patient data was restricted to authorized personnel, and all data were securely stored. To minimize the potential for stigma or discrimination, we communicated the study procedures and outcomes carefully, ensuring that participants understood the purpose of the research. We worked with clinicians to frame the results in a way that focused on improving care and avoiding any negative societal implications. |
| --- |

- 1. *Might any of the tests be experienced as invasive or culturally insensitive?*

| To minimize the risk of tests being experienced as invasive or culturally insensitive, we took great care in designing the study with cultural sensitivity in mind. We consulted with local healthcare providers and community members to ensure that all procedures were appropriate for the cultural context and aligned with patients' values and beliefs. Informed consent was obtained in a way that allowed participants to fully understand the procedures, and they were encouraged to ask questions or raise any concerns. Additionally, we ensured that the tests were as non-invasive as possible and that participants felt comfortable throughout the study. If any participant expressed discomfort, their concerns were taken seriously, and alternative approaches were considered when feasible. |
| --- |

- 1. *How did you determine that work was sensitive to traditions, restrictions, and considerations of all cultural and religious groups in the study population?*

| To ensure that the work was sensitive to the traditions, restrictions, and considerations of all cultural and religious groups in the study population, we consulted with local healthcare professionals and community leaders to understand the cultural practices and potential sensitivities that could influence participants’ willingness to engage in the study. We also considered relevant cultural factors, such as dietary restrictions and personal practices, in the study design. Informed consent was carefully tailored to ensure participants fully understood the study procedures, and we provided ample opportunity for them to ask questions or express concerns. Throughout the study, we remained responsive to feedback from participants and made adjustments when necessary to respect their cultural needs. |
| --- |

- 1. *Were biowaste and radioactive waste disposed of in accordance with local laws?*

| Yes, all biowaste and radioactive waste generated during the study were disposed of in accordance with local laws, and the disposal was handled centrally by Karolinska Institute. The institution follows established protocols for the safe handling, storage, and disposal of waste materials, ensuring compliance with environmental and safety standards. Biowaste was segregated and disposed of in designated containers, while radioactive waste was managed according to relevant safety guidelines. All waste disposal processes were regularly monitored to ensure they were conducted responsibly and in full compliance with local regulations. |
| --- |

- 1. *Were any structures built that would have impacted members of the community or the environment (such as handwashing facilities in a public space)? If so, how did you ensure that you had appropriate community buy-in?*

| N/A |
| --- |

- 1. *How might the study have impacted existing health-care resources (such as staff workloads, use of equipment that is typically employed elsewhere, or reallocation of public funds)?*

| This study could have had a limited impact on existing healthcare resources. For instance, the involvement of local healthcare staff in recruiting participants and collecting samples may have added to their workloads, though efforts were made to minimize any disruption to their routine responsibilities. Additionally, some laboratory equipment and resources may have been used for the analysis of patient samples, which might have required coordination with other departments to ensure that essential equipment was available for both the study and routine healthcare activities.No significant reallocation of public funds was necessary as the funding for the study was secured independently. |
| --- |

1. Confirm that local ethics review was sought, and please provide the approval number. If not sought, please explain why.

| Local ethics review was sought and approval was granted for this study. Samples from patients with JIA and healthy children were collected in accordance with the Declaration of Helsinki. Ethical approval was obtained from the North Ethical Committee in Stockholm, Sweden (Dnrs 2009-1139-30-4 and 2010-165-31-2 for JIA patient samples; Dnr 03-067 for healthy controls). All animal procedures were performed in compliance with protocols approved by the First Regional Ethical Committee for Animal Experiments in Kraków, Poland (approval number: 329/2022). |
| --- |

Secondary analyses

1. Have the data analysed in your study been extracted from another source, such as a national survey, rather than being directly collected by the authors of this paper?

| No. |
| --- |

If the authors of this paper were not involved in data collection, how were the findings interpreted with sufficient contextual knowledge?

The Lancet Group *believe contextual understanding is crucial for informed data analysis and interpretation.*

| As the authors were directly involved in data collection, the findings were interpreted with sufficient contextual knowledge. |
| --- |

1. Please provide the title (eg, Dr/Prof, Mr/Mrs/Ms/Mx), name, and email address of an author who can be contacted about this statement.

| **Name:** Prof. Helena Erlandsson Harris  **Email:** helena.harris@ki.se |
| --- |

1. Finally, please provide the title and name of an author from one country of study who has seen and approved this form.

| **Name:** Associate professor. Cecilia Aulin |
| --- |
